# Role of blood metabolites in mediating the effect of gut microbiome on the mutated-RAS/BRAF metastatic colorectal cancer-specific survival

**DOI:** 10.1007/s00384-024-04686-9

**Published:** 2024-07-24

**Authors:** Yaoxian Xiang, Chan Zhang, Jing Wang, Yurong Cheng, Kangjie Wang, Li Wang, Yingying Tong, Dong Yan

**Affiliations:** Department of Oncology, Beijing Luhe Hospital Affiliated to Capital Medical University, Beijing, 101149 China

**Keywords:** Mendelian randomization(MR), Gut microbiome, Metabolites, Mutated-RAS/BRAF metastatic colorectal cancer(M-mCRC), Survival, Causal relationship

## Abstract

**Background:**

Recent studies have linked alterations in the gut microbiome and metabolic disruptions to the invasive behavior and metastasis of colorectal cancer (CRC), thus affecting patient prognosis. However, the specific relationship among gut microbiome, metabolite profiles, and mutated-RAS/BRAF metastatic colorectal cancer (M-mCRC) remains unclear. Furthermore, the potential mechanisms and prognostic implications of metabolic changes induced by gut microbiome alterations in patients with M-mCRC still need to be better understood.

**Methods:**

We conducted Mendelian randomization (MR) to evaluate the causal relationship of genetically predicted 196 gut microbiome features and 1400 plasma metabolites/metabolite ratios on M-mCRC-specific survival. Additionally, we identified significant gut microbiome-metabolites/metabolite ratio associations based on M-mCRC. Metabolite information was annotated, and functional annotation and pathway enrichment analyses were performed on shared proteins corresponding to significant metabolite ratios, aiming to reveal potential mechanisms by which gut microbiome influences M-mCRC prognosis via modulation of human metabolism.

**Results:**

We identified 11 gut microbiome features and 49 known metabolites/metabolite ratios correlated with M-mCRC-specific survival. Furthermore, we identified 17 gut microbiome-metabolite/metabolite ratio associations specific to M-mCRC, involving eight lipid metabolites and three bilirubin degradation products. The shared proteins corresponding to significant metabolite ratios were predominantly localized within the integral component of the membrane and exhibited enzymatic activities such as glucuronosyltransferase and UDP-glucuronosyltransferase, crucial in processes such as glucuronidation, bile secretion, and lipid metabolism. Moreover, these proteins were significantly enriched in pathways related to ascorbate and aldarate metabolism, pentose and glucuronate interconversions, steroid hormone biosynthesis, and bile secretion.

**Conclusion:**

Our study offers novel insights into the potential mechanisms underlying the impact of the gut microbiome on the prognosis of M-mCRC. These findings serve as a meaningful reference for exploring potential therapeutic targets and strategies in the future.

**Supplementary Information:**

The online version contains supplementary material available at 10.1007/s00384-024-04686-9.

## Introduction

Colorectal cancer (CRC) is a prevalent malignancy of the digestive system and ranks as the second leading cause of cancer-related deaths [1]. An estimated 50 to 60% of cases manifest distant metastasis upon diagnosis, resulting in a poor prognosis with a 5-year survival rate ranging from merely 10 to 14% [2]. Activating missense mutations in the RAS/BRAF gene occur in about 40% of CRC patients, leading to dysregulated activation of the mitogen-activated protein kinase (MAPK) signaling pathway. This prevents patients with metastatic CRC (mCRC) from benefiting from epidermal growth factor receptor (EGFR) inhibitors such as cetuximab. Studies have demonstrated that mCRC patients with mutant RAS exhibit a worse prognosis relative to those with wild-type RAS [3–5]. At present, therapeutic options for initially unresectable RAS/BRAF-mutated microsatellite stable (MSS)-type mCRC are limited, and although clinical research has explored targeted therapeutic strategies for mutated-RAS/BRAF mCRC (M-mCRC) and treatment approaches based on various potential signaling pathways and molecules, most have failed to translate into clinical practice. The current key to research on M-mCRC is to further investigate its biology, explore new selective drugs and combinations that directly target the mutation sites, and identify metabolic pathway inhibitors that indirectly target MAPK signaling. This necessitates the identification of additional prospective prognostic and predictive biomarkers as well as potential therapeutic targets, hoping to provide a basis for formulating individualized treatment regimens and ultimately improving patient survival outcomes.

Recent studies have revealed a significant correlation between alterations in the human gut microbiome and mCRC. For instance, Bertocchi et al. found that the gut microbiome contributed to tumor metastasis by disrupting the integrity of the gut vascular barrier [6]. Yuan et al. demonstrated that alterations in the gut microbiome could induce a remodeling of the hepatic immune microenvironment by regulating the proliferation of liver Kupffer cells, thereby promoting liver metastasis of CRC [7]. However, due to the influence of confounding factors such as geographic location, dietary habits, age characteristics, treatment regimens, and specimen DNA extraction methods across various studies, the causal relationship between the abundance of specific gut microbiomes and mCRC, along with its prognostic implications for patients, remains inconclusive. Metabolites are an important factor linking the gut microbiome to CRC. Yang et al. have identified alterations in the abundance of specific gut microbiomes and plasma metabolite concentrations in CRC patients compared to healthy individuals, establishing correlations between specific gut microbiomes and metabolite profiles during the development of CRC [8]. DT et al. have revealed that the metabolite formate derived from *Fusobacterium nucleatum* promoted CRC invasion by activating AhR signal transduction [9]. These findings suggest that gut microbiomes may impact CRC development and metastasis by modulating certain metabolite levels through their effects on metabolic pathways. Metabolic reprogramming is a common characteristic observed in malignant tumors, including CRC. Dysregulated lipid metabolism-induced metabolic reshaping can lead to the accumulation of lipid droplets, thereby influencing the progression and invasion of CRC. Furthermore, it may also induce chemotherapy resistance in CRC, thereby influencing patient prognosis [10–14]. Hutton et al. found that mutations in RAS and BRAF induce metabolic reprogramming by upregulating glucose uptake and enhancing the expression of glutamine metabolism proteins [15]. Furthermore, current clinical trials are exploring potential therapeutic strategies for M-mCRC by targeting the RAS through metabolic pathways, indicating the potential influence of metabolic dysregulation on the prognosis of M-mCRC. Presently, there have been studies exploring the risk and prognostic biomarkers of CRC based on microbiomics and metabolomics. S et al. examined the metagenomics and metabolomics of 616 patients with different stages of CRC. Their findings indicated that the abundance of certain species, including *Fusobacterium nucleatum* spp, phyla *Firmicutes*, *Fusobacteria*, and *Bacteroidetes* increased with the malignancy of CRC. Metabolomic analysis revealed notable increases in branched-chain amino acids and phenylalanine in colorectal mucosal adenocarcinoma (MA), while bile acids demonstrated significant elevation in cases of multiple adenomas and/or MA. Additionally, isovaleric acid (a branched-chain fatty acid generated via bacterial fermentation from leucine17) exhibited a progressive rise in accordance with tumor staging. Consequently, these findings underscore the identification of stage-specific potential intestinal microbiota and metabolites relevant to CRC [16]. E et al. identified two potential microbial taxa, *Prevotella enoeca* and *Ruthenibacterium lactatiformans*, which exhibited higher abundances in CRC subjects with BRAFV600E mutation and BRAF wild-type, respectively. These findings proposed the potential of these microbial taxa as discerning biomarkers for the BRAF status among CRC patients [17]. However, studies on the associations between gut microbiomes and metabolites in M-mCRC are limited, and the mechanisms through which specific gut microbiomes cause alterations in metabolic pathways remain unclear. Moreover, the effects of gut microbiome-induced metabolic dysregulation on the prognosis of M-mCRC patients remain inconclusive.

Mendelian randomization (MR) is a causal inference method based on genetic variations. It involves single nucleotide polymorphisms (SNPs) strongly associated with exposure factors as instrumental variables (IVs) to infer causal relationships with outcomes. MR offers a distinct advantage by leveraging the random distribution of genotypes to effectively minimize the influence of confounding factors in observational studies, thus ensuring the robustness of causal inference.

In this study, we conducted a two-sample MR to investigate the causal impacts of gut microbiome features and plasma metabolite/metabolite ratios on M-mCRC-specific survival. Subsequently, we estimated potential causal associations between significant gut microbiome features and metabolite/metabolite ratios. Finally, we annotated metabolite information and performed functional annotation as well as pathway enrichment analyses on shared proteins corresponding to significant metabolite ratios. Our objectives were as follows: (1) to identify specific gut microbiomes and metabolite molecules associated with M-mCRC-specific survival; (2) to identify gut microbiome-metabolite associations based on M-mCRC, thereby unveiling potential mechanisms through which gut microbiome influence the prognosis of M-mCRC; (3) to uncover potential therapeutic targets for M-mCRC, thus offering insights for further investigation into treatment strategies.

## Methods

### Study design

**Fig. 1:**
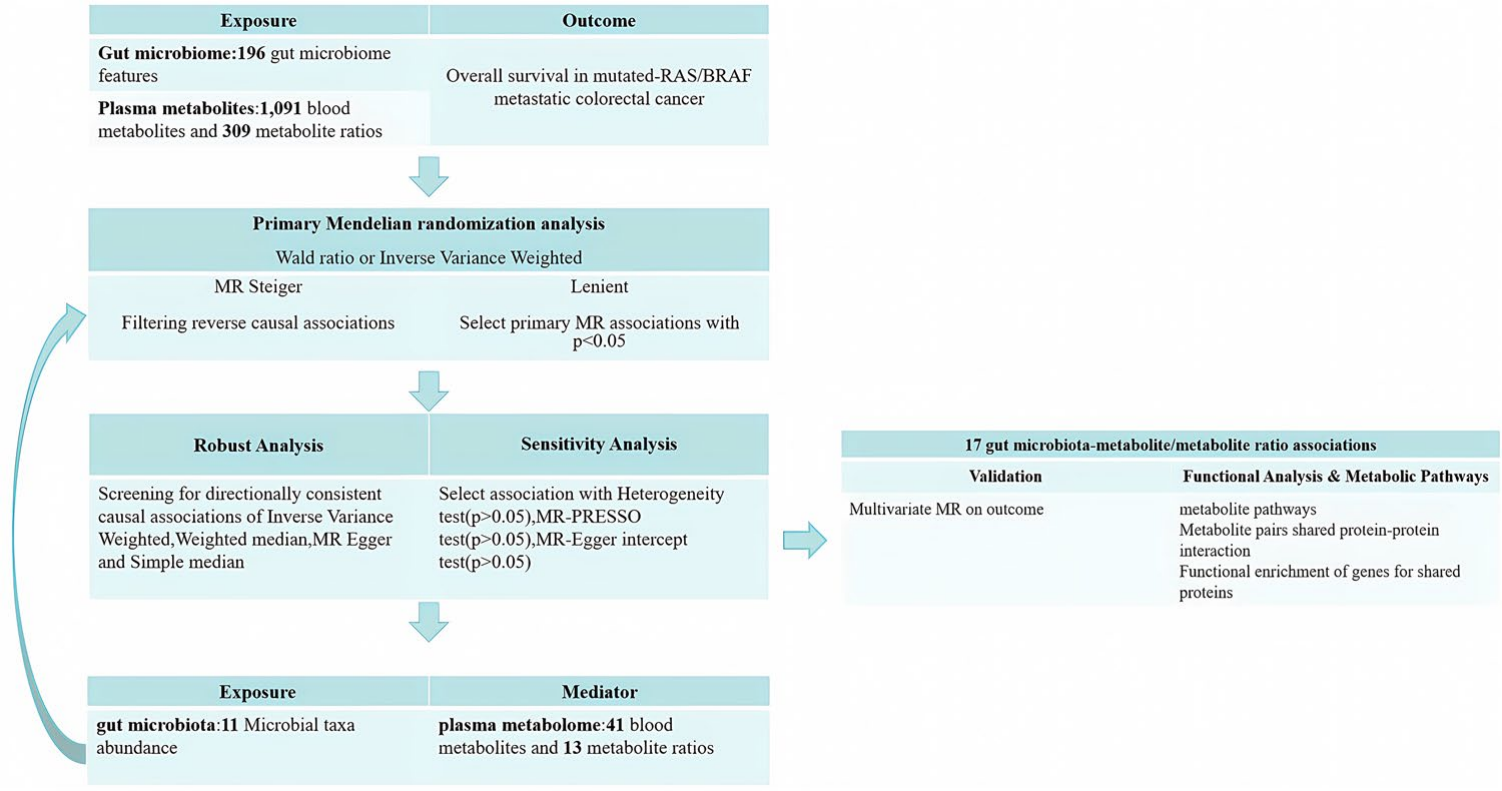
Overview of study design

The study design schematic is shown in Fig. 1. First, we screened genetic variants representing the abundance of 196 gut microbiome features and genetic variants representing levels of 1091 plasma metabolites and 309 metabolite ratios. We then selected the overall survival (OS) of M-mCRC patients in the GWAS dataset as the outcome. Next, we performed MR to (1) estimate the causal effects of 196 gut microbiome features on the outcome and identify significant gut microbiome factors, (2) estimate the causal effects of 1400 plasma metabolite/metabolite ratios on the outcome and identify significant metabolite/metabolite ratios, and (3) estimate the causal effects between significant gut microbiome and metabolites/metabolite ratios. We further individually annotated the information on significant metabolites and the shared enzymes corresponding to metabolite ratios. At last, we analyzed the interactions among these proteins, identified genes for shared proteins, and performed functional enrichment analysis. Our study design followed the STROBE-MR statement [18] (Table S1).

### Data source

Summary statistics of gut microbiomes were extracted from the publicly available GWAS dataset of the MiBioGen Consortium, encompassing 18,340 subjects across 25 cohorts and revealing a total of 196 genetically controlled gut microbiome features [19]. Genetic variants associated with plasma metabolites were obtained from the Genome-wide association study(GWAS) conducted on 8299 European participants within the Canadian Longitudinal Study on Aging (CLSA) cohort, a large-scale longitudinal study encompassing biomedical data from over 50,000 Canadian participants [20]. The genome-wide genotyping data and circulating plasma metabolite information of 8299 European participants were obtained, and finally, a total of 1091 plasma metabolites and 309 metabolite ratios involving shared enzymes under genetic control were identified [21]. The outcome summary dataset was obtained from a publicly available GWAS study, which included genotype data of 694 M-mCRC patients in the COIN [22] and COIN-B [23] clinical trials. This study integrated clinically pathological factors associated with OS (defined as the time from diagnosis to the death of patients or trial completion) to formulate regression models for survival analyses. Cox proportional hazards regression was employed to evaluate the association between genetic instruments and M-mCRC-specific survival [24].

### Selection of genetic variants

We extracted eligible genetic variants as IVs from the GWAS summary datasets of gut microbiome and plasma metabolite/metabolite ratios through the following process. First, we selected SNPs strongly associated with each exposure at the genome-wide locus significance level (*p* < 1 × 10^−5^). Second, we eliminated (1) SNPs with multiple alleles (> 2) or those located on chromosome 23, (2) SNPs with minor allele frequency (MAF) < 0.01, and (3) SNPs with linkage disequilibrium (LD) based on the criteria of *r*^2^ < 0.01 and window size > 10,000 kb with reference to the 1000 Genomes European Project. Third, we calculated F-statistic using the formula *F* = *R*^2^ × (N − 2)/(1 − *R*^2^) [25] to evaluate the efficacy of each genetic variant. A higher F-statistic indicated that the IV could better explain the variation in the dependent variable. Fourth, we determined *R*^2^ of the linear regression fit model using the formula *R*^2^ = 2 × EAF × (1 − EAF) × beta^2^/(2 × EAF × (1 − EAF) × beta^2^) + 2 × EAF × (1 − EAF) × SE × N × beta^2^ [26, 27] to quantify the degree of variance in the dependent variable explained by genetic variations. At last, we retained genetic variants with F-statistic > 10 as IVs to reduce the impact of weakly predictive IVs on MR analyses.

### MR analysis

We performed preliminary MR analyses to evaluate the causal effects of 196 gut microbiome features and 1400 plasma metabolite/metabolite ratios on the outcome, respectively. For exposures with only one IV, we employed the Wald ratio to estimate causal effects, while the inverse variance weighted (IVW) method was used for exposures with multiple IVs. IVW combines the effects of multiple IVs on outcomes to achieve an accurate estimation of causal effects through least squares estimation, thereby improving the accuracy of the estimation. We identified exposures that reached statistical significance (*p* < 0.05) and conducted supplementary MR analyses using MR-Egger, weighted median, and simple median methods. Additionally, we employed the MR Steiger test to determine the directionality of causality, mitigating the impact of reverse causal associations on MR [28]. Similarly, we evaluated the causal effect between significant gut microbiomes and plasma metabolite/metabolite ratios using the same method. Finally, we utilized multivariate MR to evaluate the independent contribution of significant gut microbiomes-metabolite/metabolite ratios to the outcome [29].

### Sensitivity analysis

To ensure the robustness of our findings, a series of sensitivity analyses were conducted for each statistically significant causal association (IVW *p* < 0.05). First, Cochran’s Q statistic was calculated by summing the squared residuals of each IV on the outcome estimates. Subsequently, a chi-square test was performed to determine the presence of variations in the impact of different IVs on the outcome. Additionally, to assess the heterogeneity of the IVs’ effects, *I*^2^ was calculated using the formula *I*^2^ = (Q-Q_df)/Q, with an *I*^2^ value of 25% or more indicating moderate to high heterogeneity [30, 31]. Moreover, we employed several approaches to mitigate the influence of IVs causally linking outcomes from confounding pathways other than exposure factors and identify and correct potential horizontal pleiotropy (HP). First, we applied the MR-Egger method to build regression models incorporating an intercept term (θ_0_) and test the significance of the Egger regression slopes to identify HP [32]. Second, we employed MR-PRESSO to (1) compute regression residuals of IVs on outcome estimates to identify HP, (2) detect and eliminate outlier IVs, and (3) generate adjusted causal estimates, thereby providing more robust MR results [33]. Third, we employed the leave-one-out method to assess the consistency of the MR results by excluding each IV and recalculating the causal effect to determine whether the causal association between exposure and outcome was significantly influenced by any of the IVs. At last, we generated scatter plots and funnel plots to visually present the estimates and their corresponding confidence intervals for each IV, with the aim of detecting potential anomalies or outliers. All statistical analyses were conducted using the TwoSampleMR and MR-PRESSO packages in R version 4.2.0 [33–35].

### Functional and metabolic pathway analysis

To investigate the potential pathways through which the gut microbiomes may impact the prognosis of M-mCRC, we further analyzed significant metabolites and shared proteins corresponding to metabolite ratios. Leveraging the Human Metabolome Database (HMDB), which provides comprehensive information on small molecule metabolites present in the human body [36], we pursued the following steps. First, we retrieved metabolite information from HMDB and annotated metabolite super and sub-pathways. Second, building upon the work of J et al., who identified 309 pairs of metabolite enzymes or transporter proteins using HMDB, we acquired information about metabolite ratios, including their shared enzymes or transporter proteins, protein types, and corresponding genes [21]. Third, we utilized the Database for Annotation, Visualization, and Integrated Discovery (DAVID) tools to annotate the cellular components (CC), biological processes (BP), and molecular functions (MF) of the shared genes, followed by pathway enrichment analysis, thereby revealing the gene functions and their roles in biological processes [37]. At last, we employed the STRING database to construct protein–protein interaction (PPI) networks of shared proteins with a minimum required interaction score of 0.4 [38].

## Results

### Association between the gut microbiome and M-mCRC-specific survival

We investigated the causal correlation between 196 gut microbiome features and the outcome at five taxonomic levels: phylum, class, order, family, and genus. We actually analyzed 187 microbiome features as IVs for 9 were not present in the outcome GWAS summary dataset. We identified 14 significant associations (*p* < 0.05) (Table S2). Further details of the IVs involved in these 14 relevant microbiomes are provided in Table S3. Notably, all IVs exhibited F-statistics greater than 10, indicating their capacity to explain the causal association between gut microbiomes and outcome. However, the results of MR Steiger did not support a unidirectional causal association of the genus *Eubacteriumeligens*, *Ruminococcus*1, and class *Alphaproteobacteria* with the outcome (Table S3). Consequently, our analysis concluded that ten gut microbiome features, including the genus *Eubacteriumhallii* (hazard ratio [HR] = 5.34, 95% confidence interval [CI] = 1.33–21.51, *p* = 0.018) and *Eubacteriumnodatum* (HR = 1.79, CI = 1.13–2.84, *p* = 0.013), were associated with worse M-mCRC-specific survival. However, the genus *Butyrivibrio* (HR = 0.56, CI = 0.34–0.93, *p* = 0.025) significantly improved patient survival (Table 1). Sensitivity analyses, as presented in Table S4 and Supplementary Figs. 1–8, confirmed the robustness of the significant causal associations (*I*^2^ < 25%, Cochran’s Q > 0.05). Both the MR-Egger intercept term and the MR-PRESSO-based test did not detect any outlier IVs or significant HP (Egger intercept *p* > 0.05, MR-PRESSO Global Test *p* > 0.05). Due to the limited IVs for the family *Oxalobacteraceae*, genus *Catenibacterium*, and *Oxalobacter*, corresponding sensitivity analyses were not performed.

**Table 1:** MR results of causal links between gut microbiome and outcome

Classification	Bacterial traits	Nsnp	Methods	HR (95% CI)	*p*-value
Family	Oxalobacteraceae.id.2966	1	Wald ratio	3.45 (1.03, 11.59)	0.0454
	Veillonellaceae.id.2172	4	Inverse variance weighted (fixed effects)	8.04 (2.53, 25.56)	0.0004
			Inverse variance weighted (multiplicative random effects)	8.04 (2.73, 23.70)	0.0002
			Weighted median	9.76 (2.07, 46.07)	0.0040
			MR Egger	0.58 (0.00, 504.24)	0.8907
			Simple median	12.27 (2.72, 55.26)	0.0011
Genus	Eubacteriumhalliigroup.id.11338	3	Inverse variance weighted (fixed effects)	5.34 (1.33, 21.51)	0.0184
			Inverse variance weighted (multiplicative random effects)	5.34 (1.28, 22.23)	0.0213
			Weighted median	3.03 (0.46, 20.01)	0.2486
			MR Egger	0.04 (0.00, 724.06)	0.8603
			Simple median	3.17 (0.45, 22.23)	0.2457
	Eubacteriumnodatumgroup.id.11297	5	Inverse variance weighted (fixed effects)	1.79 (1.13, 2.84)	0.0137
			Inverse variance weighted (multiplicative random effects)	1.79 (1.03, 3.11)	0.0391
			Weighted median	1.54 (0.81, 2.95)	0.1890
			MR Egger	10.36 (1.17, 91.48)	0.1261
			Simple median	1.56 (0.79, 3.10)	0.1996
	Butyrivibrio.id.1993	5	Inverse variance weighted (fixed effects)	0.56 (0.34, 0.93)	0.0252
			Inverse variance weighted (multiplicative random effects)	0.56 (0.36, 0.88)	0.0112
			Weighted median	0.61 (0.30, 1.25)	0.1758
			MR Egger	1.34 (0.13, 13.81)	0.8218
			Simple median	0.61 (0.31, 1.20)	0.1521
	Catenibacterium.id.2153	1	Wald ratio	3.92 (1.43, 10.76)	0.0079
	Faecalibacterium.id.2057	2	Inverse variance weighted (fixed effects)	6.04 (1.02, 35.67)	0.0472
			Inverse variance weighted (multiplicative random effects)	6.04 (4.03, 9.05)	0.0000
	Olsenella.id.822	3	Inverse variance weighted (fixed effects)	2.31 (1.22, 4.37)	0.0102
			Inverse variance weighted (multiplicative random effects)	2.31 (1.64, 3.25)	0.0000
			Weighted median	2.42 (1.08, 5.43)	0.0323
			MR Egger	3.80 (0.41, 35.01)	0.4483
			Simple median	2.53 (1.09, 5.85)	0.0305
	Oxalobacter.id.2978	1	Wald ratio	3.30 (1.02, 10.61)	0.0454
	Ruminiclostridium5.id.11355	4	Inverse variance weighted (fixed effects)	5.64 (1.68, 19.01)	0.0052
			Inverse variance weighted (multiplicative random effects)	5.64 (3.51, 9.08)	0.0000
			Weighted median	6.18 (1.41, 27.02)	0.0156
			MR Egger	7.59 (0.06, 895.66)	0.4926
			Simple median	6.26 (1.47, 26.57)	0.0130
Phylum	Proteobacteria.id.2375	3	Inverse variance weighted (fixed effects)	8.29 (2.03, 33.92)	0.0033
			Inverse variance weighted (multiplicative random effects)	8.29 (4.06, 16.93)	0.0000
			Weighted median	8.97 (1.55, 51.84)	0.0143
			MR Egger	80.47 (0.12, 556.72)	0.4139
			Simple median	11.29 (1.59, 79.99)	0.0152

### Association between plasma metabolite/metabolite ratio and M-mCRC-specific survival

**Fig. 2:**
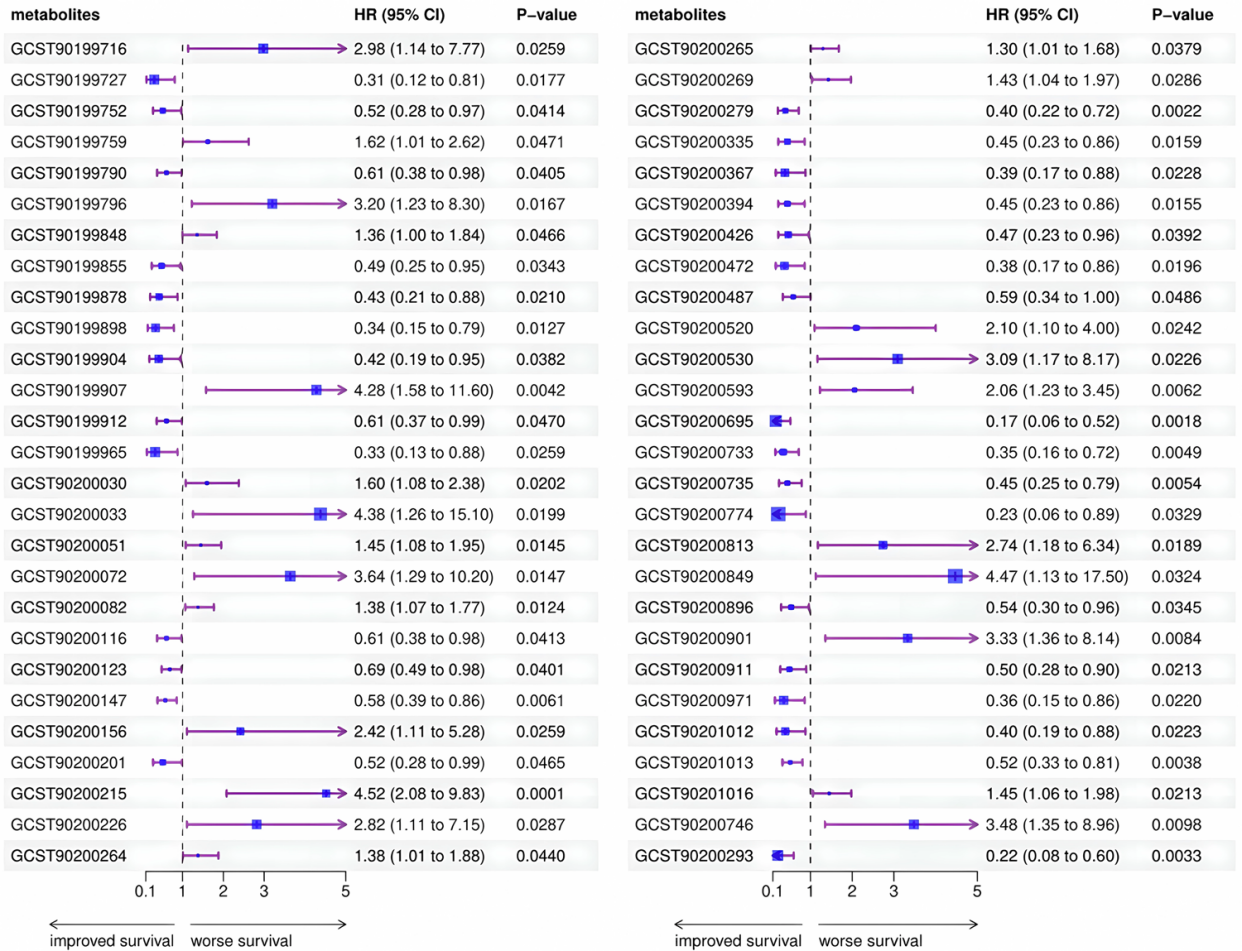
Association of genetically predicted plasma metabolite/metabolite ratios with M-mCRC-specific survival

We selected eligible genetic variants as IVs for 1091 plasma metabolites and 309 metabolite ratios and performed preliminary MR analyses. After excluding metabolites lacking IV-related information in the outcome dataset, we conducted an analysis involving 1376 metabolite-outcome associations. From this analysis, we identified 54 metabolites/metabolite ratios, of which 49 were known to have a causal association with the outcome. The remaining metabolites, X-11850, X-11880, X-13007, X-17335, and X-21834, were unknown. Among the identified associations, 20 plasma metabolites and eight metabolite ratios were positively associated with M-mCRC-specific survival (HR < 1, *p* < 0.05), while 16 plasma metabolites and five metabolite ratios were negatively associated with M-mCRC-specific survival (HR > 1, *p* < 0.05). Notably, 18 metabolites were involved in lipid metabolic pathways and 6 in amino acid metabolic pathways, highlighting the potential influence of lipids and amino acids on the prognosis of M-mCRC. Additionally, succinylcarnitine may impact energy metabolism by participating in the tricarboxylic acid cycle (TCA), whereas inosine and 2′-o-methyluridine may affect nucleotide metabolism by participating in purine and pyrimidine metabolic pathways. These findings suggest a potential role of energy and nucleotide metabolism in the progression of M-mCRC (Table S6 and Fig. 2). Supplementary Table S5 presents correlation results for all 1376 metabolites. Tables S7–8 show IVs for all 54 significant metabolites/metabolite ratios, with F-statistic > 10, indicating robust causal associations without the potential for reverse causal associations or heterogeneity.

### Association between the gut microbiome and plasma metabolite/metabolite ratio based on M-mCRC

We analyzed the 539 pairs causal correlation between eleven gut microbiomes and 49 plasma metabolite/metabolite ratios revealed in the aforementioned studies and identified 17 significant associations between gut microbiomes and metabolite/metabolite ratios, including eight products related to lipid metabolism pathways, three products related to bilirubin degradation, and one additional compound. The genus *Eubacteriumhallii* (OR = 1.18, CI = 1.01–1.38, *p* = 0.0324) and *Oxalobacter* (OR = 1.19, CI = 1.07–1.32, *p* = 0.0012) were associated with increased lipid metabolites concentration, while genus *Butyrivibrio* (OR = 0.90, CI = 0.84–0.97, *p* = 0.0079), *Catenibacterium* (OR = 0.85, CI = 0.73–0.98, *p* = 0.0233), and *Faecalibacterium* (OR = 0.79, CI = 0.67–0.94, *p* = 0.0080) and phylum *Proteobacteria* (OR = 0.80, CI = 0.68–0.95, *p* = 0.0123) exhibited negative effects on lipid metabolites. Moreover, genus *Ruminiclostridium* was negatively correlated with two lipid metabolites, sphingolipids (OR = 0.81, CI = 0.67–0.98, *p* = 0.0278) and ceramides (OR = 0.78, CI = 0.63–0.97, *p* = 0.0226) (Table 2). Further analysis of 8 lipid metabolites revealed associations with M-mCRC-specific survival. For instance, 10-undecenoate (a medium-chain fatty acid) (HR = 2.98, CI = 1.14–7.77, *p* = 0.0259); sphingomyelin (d18:2/23:0, d18:1/23:1, d17:1/24:1) (HR = 4.38, CI = 1.26–15.15, *p* = 0.0199); and 1-palmitoyl-2-dihomo-linolenoyl-GPC (a phosphatidylcholine) (HR = 1.45, CI = 1.08–1.95, *p* = 0.0145) were associated with worse M-mCRC-specific survival, while 1-oleoyl-GPC (HR = 0.31, CI = 0.12–0.81, *p* = 0.0177); 2-palmitoleoyl-GPC (both lysophospholipid) (HR = 0.61, CI = 0.38–0.98, *p* = 0.0405); 5alpha-androstan-3beta, 17beta-diol monosulfate (an Androgenic Steroid) (HR = 0.49, CI = 0.25–0.95, *p* = 0.0343); ceramide (d18:1/14:0, d16:1/16:0) (HR = 0.61, CI = 0.38–0.98, *p* = 0.0413); and stearoyl sphingomyelin (d18:1/18:0) (both Sphingomyelins) (HR = 0.45, CI = 0.23–0.86, *p* = 0.0159) were associated with improved survival. These findings suggested that gut microbiomes could impact the prognosis of M-mCRC by participating in lipid metabolism. Notably, genus *Olsenella* was positively correlated with the concentrations of the three bilirubin degradation products (OR > 1, *p* < 0.05), and higher *Olsenella* abundance and bilirubin degradation products were both correlated with poor M-mCRC-specific survival (HR > 1, *p* < 0.05), suggesting a potential association between genus *Olsenella* and poor survival of M-mCRC by promoting bilirubin degradation (Table S6). Table S9 displays the correlation results for the 539 association pairs. Tables S10–11 and Supplementary Figs. 9–25 provide information on IVs, Steiger test results, and sensitivity analysis results for the 17 significant gut microbiome-metabolite associations.

**Table 2:** MR results of causal links between gut microbiome and metabolites/metabolite ratios based on M-mCRC

Gut microbiome	ID	Metabolites/Metabolite ratios	Super_pathway	Sub_pathway	Nsnp	Methods	OR(95%CI)	*p*−value
genus.Eubacteriumhalliigroup.id.11338	GCST90199716	10-undecenoate (11:1n1) levels	Lipid	Medium Chain Fatty Acid	12	Inverse variance weighted (fixed effects)	1.18(1.01,1.38)	0.0324
					12	Inverse variance weighted (multiplicative random effects)	1.18(1.01,1.38)	0.0321
					12	Weighted median	1.20(0.97,1.49)	0.0953
					12	MR Egger	1.09(0.77,1.52)	0.6442
					12	Simple median	1.24(1.02,1.51)	0.0329
	GCST90200901	Ornithine to phosphate ratio	Amino Acid / Energy	/	12	Inverse variance weighted (fixed effects)	1.30(1.11,1.50)	0.0007
					12	Inverse variance weighted (multiplicative random effects)	1.30(1.12,1.50)	0.0005
					12	Weighted median	1.16(0.94,1.43)	0.1709
					12	MR Egger	1.34(0.97,1.85)	0.1100
					12	Simple median	1.16(0.94,1.42)	0.1652
genus.Butyrivibrio.id.1993	GCST90199727	1-oleoyl-GPC (18:1) levels	Lipid	Lysophospholipid	13	Inverse variance weighted (fixed effects)	0.90(0.84,0.97)	0.0079
					13	Inverse variance weighted (multiplicative random effects)	0.90(0.84,0.98)	0.0110
					13	Weighted median	0.93(0.83,1.03)	0.1819
					13	MR Egger	0.98(0.69,1.39)	0.9046
					13	Simple median	0.93(0.84,1.03)	0.1707
	GCST90200971	Leucine to phosphate ratio	Amino Acid / Energy	/	13	Inverse variance weighted (fixed effects)	0.92(0.86,0.99)	0.0221
					13	Inverse variance weighted (multiplicative random effects)	0.92(0.87,0.98)	0.0089
					13	Weighted median	0.92(0.84,1.01)	0.0873
					13	MR Egger	0.71(0.53,0.96)	0.0476
					13	Simple median	0.92(0.83,1.02)	0.1000
	GCST90201012	3-methyl-2-oxovalerate to 3-methyl-2-oxobutyrate ratio	Amino Acid / Amino Acid	/	13	Inverse variance weighted (fixed effects)	0.91(0.85,0.98)	0.0151
					13	Inverse variance weighted (multiplicative random effects)	0.91(0.85,0.98)	0.0110
					13	Weighted median	0.94(0.85,1.05)	0.2593
					13	MR Egger	0.90(0.65,1.24)	0.5329
					13	Simple median	0.94(0.85,1.04)	0.2243
	GCST90201016	Bilirubin (Z,Z) to etiocholanolone glucuronide ratio	Cofactors and Vitamins / Lipid	/	13	Inverse variance weighted (fixed effects)	0.87(0.81,0.94)	0.0004
					13	Inverse variance weighted (multiplicative random effects)	0.87(0.80,0.94)	0.0009
					13	Weighted median	0.87(0.78,0.97)	0.0097
					13	MR Egger	0.73(0.51,1.04)	0.1122
					13	Simple median	0.87(0.78,0.97)	0.0110
genus.Catenibacterium.id.2153	GCST90199790	2-palmitoleoyl-GPC (16:1) levels	Lipid	Lysophospholipid	4	Inverse variance weighted (fixed effects)	0.85(0.73,0.98)	0.0233
					4	Inverse variance weighted (multiplicative random effects)	0.85(0.78,0.92)	0.0001
					4	Weighted median	0.86(0.73,1.02)	0.0768
					4	MR Egger	1.03(0.17,6.31)	0.9760
					4	Simple median	0.86(0.73,1.01)	0.0669
genus.Oxalobacter.id.2978	GCST90199855	5alpha-androstan-3beta,17beta-diol monosulfate (2) levels	Lipid	Androgenic Steroids	8	Inverse variance weighted (fixed effects)	1.19(1.07,1.32)	0.0012
					8	Inverse variance weighted (multiplicative random effects)	1.19(1.05,1.34)	0.0051
					8	Weighted median	1.12(0.97,1.29)	0.1267
					8	MR Egger	1.38(0.78,2.46)	0.3102
					8	Simple median	1.13(0.98,1.30)	0.0891
genus.Ruminiclostridium5.id.11355	GCST90200033	Sphingomyelin (d18:2/23:0, d18:1/23:1, d17:1/24:1) levels	Lipid	Sphingomyelins	6	Inverse variance weighted (fixed effects)	0.81(0.65,1.01)	0.0670
					6	Inverse variance weighted (multiplicative random effects)	0.81(0.67,0.98)	0.0278
					6	Weighted median	0.87(0.66,1.15)	0.3329
					6	MR Egger	0.95(0.37,2.42)	0.9129
					6	Simple median	0.84(0.63,1.12)	0.2261
	GCST90200116	Ceramide (d18:1/14:0, d16:1/16:0) levels	Lipid	Ceramides	6	Inverse variance weighted (fixed effects)	0.78(0.59,1.02)	0.0683
					6	Inverse variance weighted (multiplicative random effects)	0.78(0.63,0.97)	0.0226
					6	Weighted median	0.73(0.52,1.03)	0.0760
					6	MR Egger	1.49(0.48,4.60)	0.5301
					6	Simple median	0.71(0.50,1.02)	0.0658
phylum.Proteobacteria.id.2375	GCST90200051	1-palmitoyl-2-dihomo-linolenoyl-GPC (16:0/20:3n3 or 6) levels	Lipid	Phosphatidylcholine (PC)	7	Inverse variance weighted (fixed effects)	0.80(0.65,1.00)	0.0506
					7	Inverse variance weighted (multiplicative random effects)	0.80(0.68,0.95)	0.0123
					7	Weighted median	0.78(0.59,1.04)	0.0928
					7	MR Egger	0.54(0.30,0.99)	0.1032
					7	Simple median	0.79(0.59,1.06)	0.1124
	GCST90200226	5-hydroxy-2-methylpyridine sulfate levels	Xenobiotics	Chemical	7	Inverse variance weighted (fixed effects)	0.74(0.58,0.94)	0.0134
					7	Inverse variance weighted (multiplicative random effects)	0.74(0.59,0.92)	0.0064
					7	Weighted median	0.76(0.55,1.06)	0.1047
					7	MR Egger	0.69(0.36,1.34)	0.3201
					7	Simple median	0.76(0.54,1.06)	0.1078
genus.Olsenella.id.822	GCST90200264	Bilirubin degradation product, C17H20N2O5 (2) levels	Partially Characterized Molecules	Partially Characterized Molecules	8	Inverse variance weighted (fixed effects)	1.14(1.03,1.26)	0.0130
					8	Inverse variance weighted (multiplicative random effects)	1.14(1.03,1.26)	0.0136
					8	Weighted median	1.08(0.94,1.23)	0.2745
					8	MR Egger	1.04(0.67,1.60)	0.8750
					8	Simple median	1.08(0.95,1.24)	0.2449
	GCST90200265	Bilirubin degradation product, C16H18N2O5 (4) levels	Partially Characterized Molecules	Partially Characterized Molecules	8	Inverse variance weighted (fixed effects)	1.12(1.01,1.24)	0.0270
					8	Inverse variance weighted (multiplicative random effects)	1.12(1.02,1.23)	0.0169
					8	Weighted median	1.15(1.00,1.31)	0.0473
					8	MR Egger	1.26(0.84,1.90)	0.3010
					8	Simple median	1.15(1.00,1.31)	0.0421
	GCST90200269	Bilirubin degradation product, C17H20N2O5 (1) levels	Partially Characterized Molecules	Partially Characterized Molecules	8	Inverse variance weighted (fixed effects)	1.13(1.02,1.25)	0.0168
					8	Inverse variance weighted (multiplicative random effects)	1.13(1.02,1.25)	0.0159
					8	Weighted median	1.07(0.94,1.23)	0.2988
					8	MR Egger	1.05(0.68,1.61)	0.8388
					8	Simple median	1.08(0.95,1.24)	0.2352
genus.Faecalibacterium.id.2057	GCST90200335	Stearoyl sphingomyelin (d18:1/18:0) levels	Lipid	Sphingomyelins	7	Inverse variance weighted (fixed effects)	0.79(0.67,0.94)	0.0080
					7	Inverse variance weighted (multiplicative random effects)	0.79(0.63,0.99)	0.0415
					7	Weighted median	0.87(0.68,1.11)	0.2571
					7	MR Egger	0.97(0.66,1.44)	0.8984
					7	Simple median	0.72(0.54,0.97)	0.0313
	GCST90201012	3-methyl-2-oxovalerate to 3-methyl-2-oxobutyrate ratio	Amino Acid / Amino Acid	/	7	Inverse variance weighted (fixed effects)	1.32(1.11,1.57)	0.0019
					7	Inverse variance weighted (multiplicative random effects)	1.32(1.07,1.63)	0.0099
					7	Weighted median	1.38(1.07,1.77)	0.0123
					7	MR Egger	1.27(0.84,1.93)	0.3047
					7	Simple median	1.37(1.03,1.81)	0.0307

### Independent effects of gut microbiome-metabolite/metabolite ratios on M-mCRC-specific survival

We performed multivariate MR to estimate the independent effects of the aforementioned 17 pairs of gut microbiomes and metabolite/metabolite ratios on M-mCRC-specific survival. As shown in Table 3, the genus *Ruminiclostridium* and *Olsenella* and the ornithine-to-phosphate ratio were significantly associated with worse M-mCRC-specific survival (*β* > 0, *p* < 0.05). Conversely, only stearoyl sphingomyelin (*β* =  − 1.54, CI =  − 2.82 to − 0.25, *p* = 0.033) was positively associated with M-mCRC-specific survival. Notably, after removing the effect of the gut microbiome, the effects of ceramide (d18:1/14:0, d16:1/16:0), 1-palmitoyl-2-dihomo-linolenoyl-GPC, and 5-hydroxy-2-methylpyridine sulfate on outcome were inconsistent with the original effects, although not statistically significant. We hypothesized that this inconsistency might be attributed to the inhibition of these metabolites by the gut microbiome. Although the residual independent impacts of gut microbiome and metabolites on the outcome did not attain statistical significance, they consistently exhibited the same trend as the initial effect.

**Table 3:** Multivariate MR estimates of the effect of gut microbiome-metabolite/metabolite ratio on M-mCRC-specific survival

Exposure	Metabolites/metabolite ratio	Method	β (95% CI)	P	Cochran Q	PHeterogeneity	Egger intercept	Pintercept
		IVW	0.32 (− 0.81, 1.44)	0.583	8.488	0.387	8.104	0.324
			0.87 (0.07, 1.67)	0.033				
genus.Eubacteriumhalliigroup.id.11338/GCST90200901	Ornithine to phosphate ratio	MR Egger	0.71 (− 1.48, 2.90)	0.526	8.280	0.309		
			0.82(-0.06,1.69)	0.068				
		Weighted median	1.05 (− 0.71, 2.81)	0.242				
			0.84 (− 0.25, 1.93)	0.131				
		IVW	0.31 (− 0.93, 1.54)	0.629	8.745	0.272	8.327	0.215
			0.87 (− 0.06, 1.81)	0.067				
genus.Eubacteriumhalliigroup.id.11338/GCST90199716	10-undecenoate (11:1n1) levels	MR Egger	0.47 (− 1.79, 2.73)	0.683	8.699	0.191		
			0.89 (− 0.14, 1.93)	0.091				
		Weighted median	0.69 (− 1.05, 2.42)	0.44				
			1.10 (− 0.18, 2.38)	0.093				
		IVW	− 0.29 (− 0.94, 0.35)	0.374	18.043	0.021	17.825	0.013
			0.06 (− 1.39, 1.50)	0.94				
genus.Butyrivibrio.id.1993/GCST90199727	1-oleoyl-GPC (18:1) levels	MR Egger	− 0.43 (− 1.84, 0.98)	0.551	17.924	0.012		
			0.02 (− 1.56, 1.60)	0.982				
		Weighted median	− 0.40 (− 1.06, 0.27)	0.239				
			− 0.80 (− 2.28, 0.67)	0.285				
		IVW	− 0.29 (− 0.83, 0.24)	0.282	15.112	0.088	14.755	0.064
			− 0.46 (− 1.78, 0.85)	0.489				
genus.Butyrivibrio.id.1993/GCST90200971	Leucine to phosphate ratio	MR Egger	0.07 (− 0.93, 1.07)	0.888	13.843	0.086		
			− 0.24 (− 1.67, 1.19)	0.744				
		Weighted median	− 0.62 (− 1.27, 0.03)	0.062				
			− 1.01 (− 2.38, 0.36)	0.149				
		IVW	− 0.26 (− 0.77, 0.25)	0.32	15.440	0.117	14.758	0.098
			− 0.78 (− 1.90, 0.35)	0.177				
genus.Butyrivibrio.id.1993/GCST90201012	3-methyl-2-oxovalerate to 3-methyl-2-oxobutyrate ratio	MR Egger	− 0.77 (− 1.92, 0.38)	0.191	13.986	0.123		
			− 0.90 (− 2.06, 0.26)	0.128				
		Weighted median	− 0.49 (− 1.15, 0.18)	0.149				
			− 0.13 (− 1.46, 1.21)	0.852				
		IVW	− 0.20 (− 0.77, 0.36)	0.479	14.087	0.080	13.577	0.059
			0.68 (− 0.50, 1.86)	0.258				
genus.Butyrivibrio.id.1993/GCST90201016	Bilirubin (Z,Z) to etiocholanolone glucuronide ratio	MR Egger	0.48 (− 0.72, 1.67)	0.432	11.495	0.118		
			0.50 (− 0.67, 1.67)	0.404				
		Weighted median	− 0.39 (− 1.06, 0.28)	0.254				
			0.79 (− 0.61, 2.19)	0.268				
		IVW	0.60 (− 0.26, 1.46)	0.174	5.514	0.239	5.162	0.160
			− 0.36 (− 1.30, 0.59)	0.458				
genus.Catenibacterium.id.2153/GCST90199790	2-palmitoleoyl-GPC (16:1) levels	MR Egger	0.91 (− 0.58, 2.41)	0.231	5.028	0.170		
			− 0.18 (− 1.41, 1.05)	0.775				
		Weighted median	0.76 (− 0.66, 2.19)	0.293				
			− 0.30 (− 1.40, 0.81)	0.599				
		IVW	0.21 (− 0.56, 0.98)	0.59	3.821	0.576	3.684	0.451
			− 0.70 (− 1.50, 0.10)	0.085				
genus.Oxalobacter.id.2978/GCST90199855	5alpha-androstan-3beta, 17beta-diol monosulfate (2) levels	MR Egger	0.58 (− 0.43, 1.58)	0.26	2.592	0.628		
			− 0.76 (− 1.56, 0.05)	0.065				
		Weighted median	0.02 (− 1.16, 1.20)	0.972				
			− 0.44 (− 1.56, 0.67)	0.436				
		IVW	1.49 (0.48, 2.50)	0.004	10.692	0.297	9.831	0.277
			− 0.62 (− 1.61, 0.38)	0.225				
genus.Ruminiclostridium5.id.11355/GCST90200033	Sphingomyelin (d18:2/23:0, d18:1/23:1, d17:1/24:1) levels	MR Egger	1.45 (− 0.32, 3.23)	0.108	10.689	0.220		
			− 0.62 (− 1.68, 0.44)	0.252				
		Weighted median	1.25 (− 0.07, 2.57)	0.063				
			0.29 (− 1.33, 1.91)	0.727				
		IVW	1.41 (0.49, 2.33)	0.003	3.026	0.990	2.846	0.985
			0.30 (− 0.345, 0.96)	0.36				
genus.Ruminiclostridium5.id.11355/GCST90200116	Ceramide (d18:1/14:0, d16:1/16:0) levels	MR Egger	1.43 (− 0.04, 2.89)	0.056	3.025	0.981		
			0.31 (− 0.36, 0.97)	0.365				
		Weighted median	1.19 (− 0.05, 2.43)	0.06				
			0.34 (− 0.56, 1.24)	0.46				
		IVW	0.49 (− 0.50, 1.48)	0.332	14.295	0.353	14.154	0.291
			− 0.22 (− 0.86, 0.43)	0.51				
phylum.Proteobacteria.id.2375/ − GCST90200051	1-palmitoyl-2-dihomo-linolenoyl-GPC (16:0/20:3n3 or 6) levels	MR Egger	1.13 (− 0.26, 2.52)	0.111	12.626	0.397		
			− 0.29 (− 0.93, 0.35)	0.378				
		Weighted median	0.47 (− 1.11, 2.05)	0.561				
			− 0.08 (− 0.95, 0.79)	0.856				
		IVW	0.13 (− 1.34, 1.60)	0.858	13.631	0.058	13.621	0.034
			− 0.05 (− 1.29, 1.19)	0.937				
phylum.Proteobacteria.id.2375/GCST90200226	5-hydroxy-2-methylpyridine sulfate levels	MR Egger	− 3.89 (− 8.11, 0.34)	0.071	8.336	0.215		
			− 1.61 (− 3.48, 0.27)	0.094				
		Weighted median	− 0.04 (− 1.75, 1.66)	0.961				
			− 1.18 (− 2.95, 0.59)	0.19				
		IVW	0.99 (0.20, 1.78)	0.014	1.645	0.649	1.421	0.491
			0.32 (− 0.67, 1.31)	0.525				
genus.Olsenella.id.822/GCST90200264	Bilirubin degradation product, C17H20N2O5 (2) levels	MR Egger	1.70 (− 0.36, 3.76)	0.107	1.113	0.573		
			− 0.43 (− 2.68, 1.82)	0.706				
		Weighted median	0.94 (− 0.30, 2.19)	0.137				
			0.23 (− 1.26, 1.73)	0.76				
		IVW	0.68 (0.06, 1.30)	0.031	2.851	0.827	2.675	0.750
			− 0.01 (− 0.76, 0.75)	0.983				
genus.Olsenella.id.822/GCST90200265	Bilirubin degradation product, C16H18N2O5 (4) levels	MR Egger	1.22 (0.18, 2.26)	0.021	1.255	0.940		
			− 0.11 (− 0.88, 0.66)	0.778				
		Weighted median	0.39 (− 0.52, 1.30)	0.403				
			0.10 (− 0.92, 1.11)	0.851				
		IVW	0.10 (0.20, 1.79)	0.014	2.013	0.733	1.763	0.623
			0.19 (− 0.69, 1.06)	0.68				
genus.Olsenella.id.822/GCST90200269	Bilirubin degradation product, C17H20N2O5 (1) levels	MR Egger	1.29 (− 0.56, 3.15)	0.171	1.891	0.595		
			− 0.16 (− 2.31, 1.98)	0.881				
		Weighted median	0.93 (− 0.26, 2.11)	0.125				
			− 0.22 (− 1.56, 1.12)	0.746				
		IVW	1.23 (− 0.64, 3.09)	0.197	6.759	0.149	6.359	0.095
			− 0.56 (− 1.86, 0.74)	0.4				
genus.Faecalibacterium.id.2057/GCST90200335	Stearoyl sphingomyelin (d18:1/18:0) levels	MR Egger	− 2.03 (− 5.07, 1.01)	0.191	1.109	0.775		
			− 1.54 (− 2.82, − 0.25)	0.019				
		Weighted median	1.52 (− 0.54, 3.58)	0.149				
			− 0.16 (− 1.77, 1.45)	0.846				
		IVW	1.26 (− 0.56, 3.08)	0.174	3.403	0.334	3.145	0.208
			− 0.76 (− 1.88, 0.35)	0.179				
genus.Faecalibacterium.id.2057/GCST90201012	3-methyl-2-oxovalerate to 3-methyl-2-oxobutyrate ratio	MR Egger	0.08 (− 3.81, 3.98)	0.967	2.749	0.253		
			− 0.56 (− 1.92, 0.81)	0.425				
		Weighted median	2.45 (− 0.56, 5.45)	0.11				
			− 0.72 (− 2.20, 0.77)	0.343				

### Analysis of the functional roles and metabolic pathways associated with shared proteins corresponding to significant metabolite ratios

**Fig. 3:**
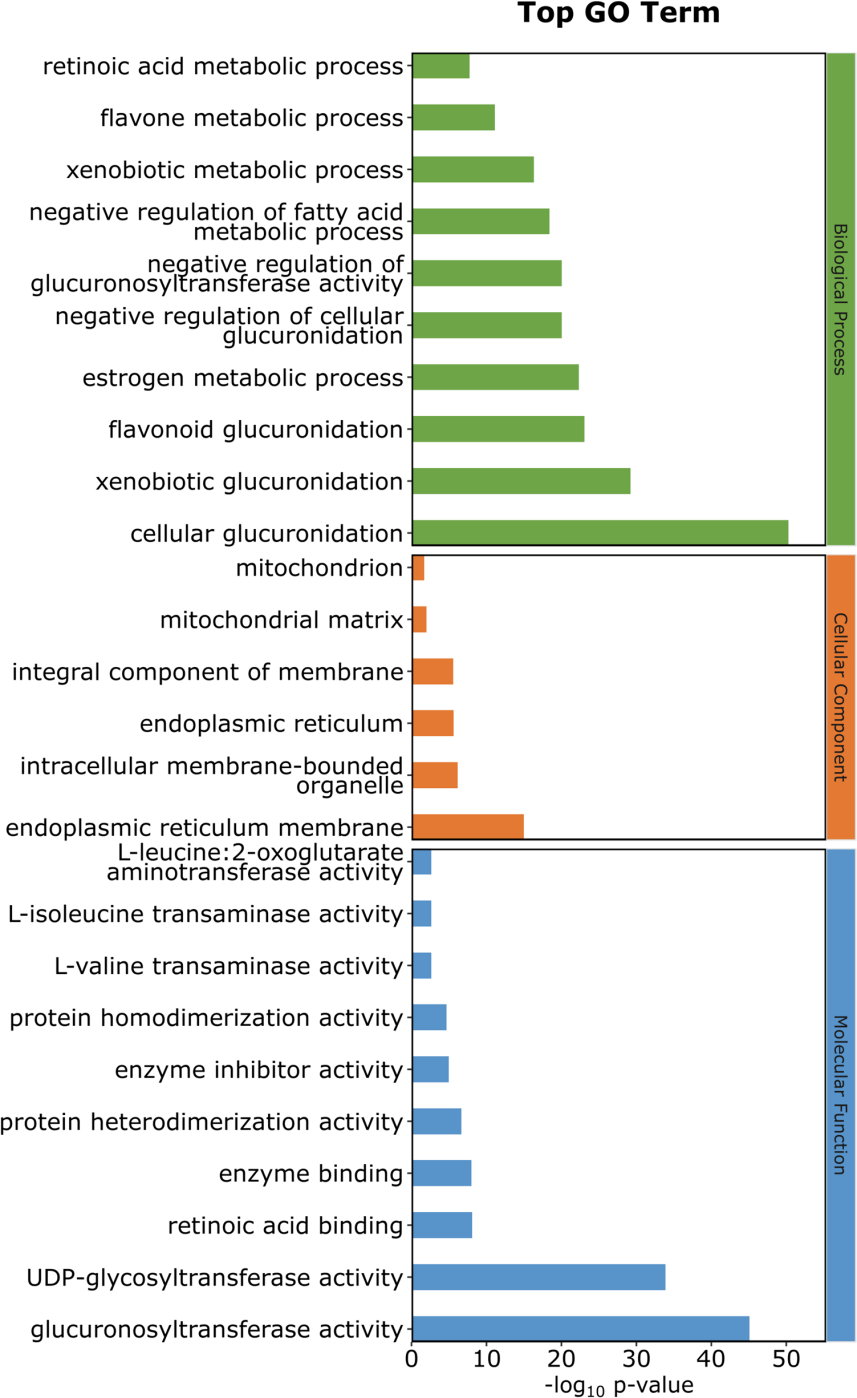
BP, CC and MF annotation analysis of gene for shared protein of significant metabolite ratios

**Fig. 4:**
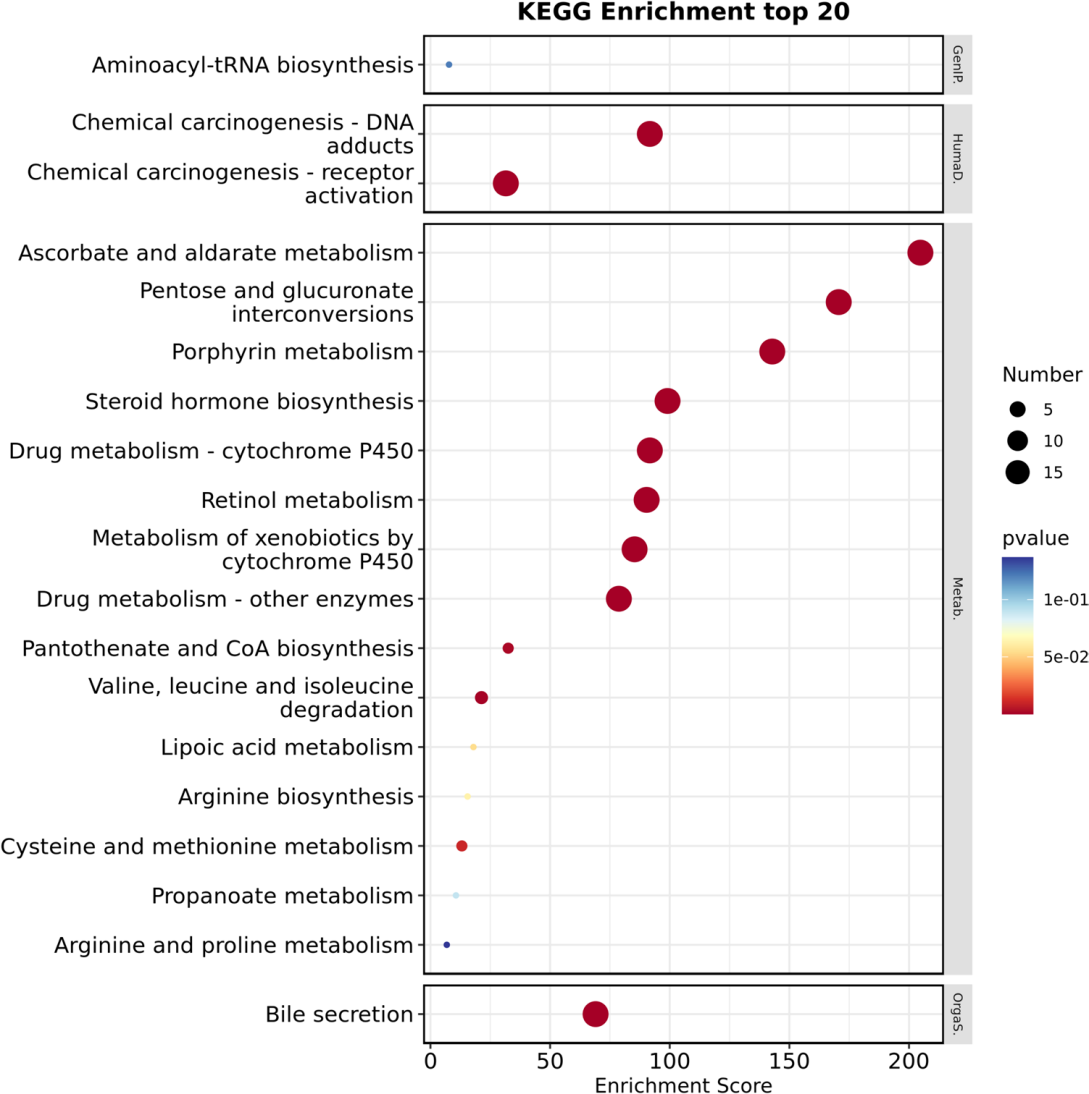
KEGG pathways annotation analysis of gene for shared protein of significant metabolite ratios. Classification level: Genetic Information Processing (GenIP), Human Diseases (HumaD), Metabolism (Metab)

**Fig. 5:**
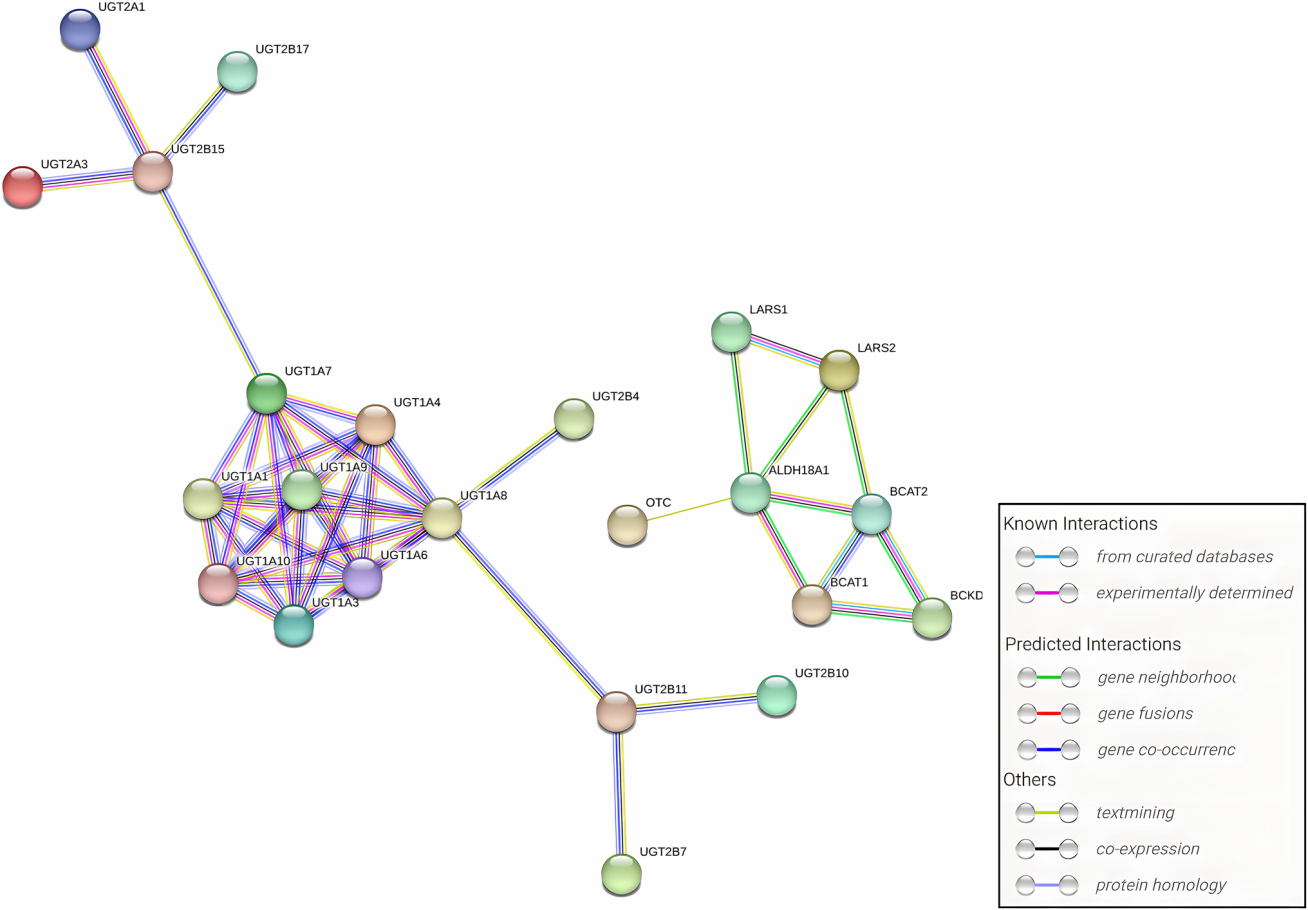
PPI network of interactions between shared protein genes

We obtained shared proteins and genes for shared proteins of significant metabolite ratios and performed functional annotation of the shared genes using the DAVID tool (Table 4). The shared proteins were mainly localized in the endoplasmic reticulum membrane, with a lesser presence in the intracellular membrane-bounded organelle and integral components of membranes. They demonstrated diverse enzyme activities, including glucuronosyltransferase and UDP-glycosyltransferase, involved in metabolic processes such as glucuronidation, steroid and lipid metabolism, and branched-chain amino acid metabolism. Moreover, they exhibited significant enrichment in pathways such as ascorbate and aldarate metabolism, pentose and glucuronate interconversions, porphyrin metabolism, steroid hormone biosynthesis, and bile secretion (Figs. 3–4 and Tables S12–13). These findings suggest that the gut microbiome may affect M-mCRC-specific survival by regulating the enzymatic activities of organelle membrane proteins involved in glucuronidation and the metabolism of a variety of substances such as steroids, lipids, and amino acids. The PPI network constructed based on STRING revealed interactions between genes encoding shared proteins (Fig. 5).

**Table 4:** Information of significant metabolite ratios

ID	Metabolite ratios	Super pathways	Gene for shared protein	Protein names	Protein type
GCST90200901	Ornithine/phosphate	Amino acid/energy	ALDH18A1;OTC	Delta-1-pyrroline-5-carboxylate synthase; Ornithine carbamoyltransferase	Enzyme; enzyme
GCST90200971	Leucine/phosphate	Amino acid/energy	LARS2;LARS	Probable leucine–tRNA ligase; Leucine–tRNA ligase	Enzyme; enzyme
GCST90201016	Bilirubin (Z,Z)/etiocholanolone glucuronide	Cofactors and vitamins/lipid	UGT2B28;UGT2B4;UGT1A4;UGT2B10; UGT2B7;UGT2B15;UGT2A1;UGT1A1; UGT1A9;UGT1A8;UGT1A3;UGT1A10; UGT2B17;UGT1A6;UGT1A5;UGT2B11; UGT1A7; UGT2A3	UDP-glucuronosyltransferase 2B28; 2B4; 1–4;2B10;2B7;2B15;2A1;1–1;1–9;1–8; 1–3;1–10;2B17;1–6;1–5;2B11;1–7;2A3	Enzyme; enzyme; Enzyme; Enzyme; enzyme; Enzyme; Enzyme; enzyme; Enzyme; Enzyme; enzyme; Enzyme; Enzyme; enzyme; Enzyme; Enzyme; enzyme; Enzyme
GCST90201012	3-Methyl-2-oxovalerate/3-methyl-2-oxobutyrate	Amino acid/amino acid	BCKDHB;BCAT1;BCAT2	2-oxoisovalerate dehydrogenase subunit beta; Branched-chain-amino-acid aminotransferase; Branched-chain-amino-acid aminotransferase	Enzyme; enzyme; Enzyme

## Discussion

In this study, MR analyses were employed to evaluate the potential causal effects of genetically predicted gut microbiome and blood metabolites on M-mCRC-specific survival and identify the gut microbiome-metabolite/metabolite ratios associations based on M-mCRC. Moreover, functional annotation and pathway enrichment analyses of shared proteins corresponding to significant metabolite ratios were performed to reveal the potential mechanisms underlying the impacts of the gut microbiome on M-mCRC-specific survival through modulation of human metabolism. In total, we identified significant correlations between eleven gut microbiome features and 49 known metabolites/metabolite ratios with M-mCRC-specific survival. Moreover, correlation analyses of significant gut microbiome and metabolites/metabolite ratios revealed 17 potential associations for gut microbiome-metabolites-M-mCRC-specific survival pathways.

Our findings unveiled a negative correlation between genus *Eubacteriumhallii* and M-mCRC-specific survival via positively regulating the ornithine to phosphate ratio (O/P). δ-1-pyrroline-5-carboxylate synthase (P5CS) and ornithine carbamoyltransferase (OTC) were shared proteins for the O/P. MR results indicated an association between elevated O/P and worse M-mCRC-specific survival. OTC catalyzes the urea cycle, while P5CS is primarily involved in synthesizing δ-1-pyrroline5-carboxylate (P5C), an intermediate product in the conversion of proline, glutamate, and ornithine. This process interconnects the TCA cycle, urea cycle, and proline metabolism, impacting cell growth, redox homeostasis, ATP production, and immune regulation [39–41]. Previous studies have demonstrated elevated P5CS expression in various malignancies, including hepatocellular carcinoma, where it plays a pivotal role in tumor metabolic remodeling and progression, correlating with poor patient prognosis [42, 43]. Additionally, the long isoform of P5CS (P5CS.long) encoded by selective splicing of the RNA transcript is significantly up-regulated by the oncogene p53 during apoptosis of DLD-1, a type of CRC cells [44]. Despite limited literature on the correlation of genus *Eubacteriumhallii* with CRC, we hypothesized that the gut microbiome may trigger upregulation of P5CS, thereby impacting cellular functions and dysregulating various metabolic pathways, including proline metabolism. This could lead to remodeling of the tumor microenvironment, affecting the efficacy of anti-cancer treatments and ultimately impacting the prognosis of patients with M-mCRC.

Our study highlighted the genus *Butyrivibrio* as the sole microbiome associated with improved M-mCRC-specific survival. *Butyrivibrio*, a bacterium known for producing butyrate, a short-chain fatty acid, has been linked to a reduced risk of CRC [45]. Butyrate plays a crucial role in preserving the integrity of the colonic epithelial cell barrier. Moreover, it inhibits angiogenesis and metastasis of CRC cells by suppressing Sp1 activation and down-regulating Neuropilin-1 (NRP-1) expression [46, 47]. Thus, butyrate may act as a protective factor against CRC by impeding its development and progression through multiple pathways. Our study further revealed that *Butyrivibrio* and its byproduct, butyrate, potentially correlate with improved prognoses of M-mCRC by modulating human metabolism. Elevated abundance of *Butyrivibrio* exhibited a negative correlation with three metabolite ratios, indicating a potential suppression of enzymes corresponding to the metabolite ratios, including leucine-tRNA ligase (LRS), branched-chain-amino-acid aminotransferase (BCAT1, BCAT2), and various UDP-glucuronosyltransferases. Studies have demonstrated that the LRS may activate the mammalian target of the rapamycin family (mTOR) by binding to RagD GTPases. Dysregulated activation of the mTOR signaling pathway has been implicated in the development and progression of CRC [48–50]. Furthermore, researchers have identified mTORC1 inhibitors targeting LRS with specific cytotoxicity against mTORC1-overexpressing CRC cells as potential therapeutic targets for CRC [51]. In addition, clinical studies have linked hypermethylated BCAT1 after radical CRC surgery to a higher recurrence rate. Methylated BCAT1 in human plasma may serve as a potential diagnostic and prognostic indicator for CRC [52, 53]. However, the specific interactions of *Butyrivibrio* and its product butyrate with LRS, mTOR, and BCAT are currently unknown, necessitating further investigation to elucidate their potential associations. In conclusion, our study sheds light on the impact of the butyrate-producing bacterium *Butyrivibrio* on M-mCRC-specific survival and identifies potential functional targets and metabolic pathways worthy of further exploration.

Our study revealed an intriguing finding regarding another butyrate-producing bacterium, the genus *Faecalibacterium*, which exhibited an opposite effect. Several studies have highlighted its role in immune system modulation and preservation of intestinal barrier integrity [54]. Elevated abundance of *F*aecalibacterium has been associated with enhanced immune therapeutic responses in various cancers, including non-small cell lung carcinoma (NSCLC), hepatocellular carcinoma, and melanoma [55–59]. For instance, Dikeocha et al. constructed an Azoxymethane (AOM)-induced CRC model in rats and observed that *Faecalibacterium* could suppress lipid peroxidation and CRC cell proliferation in colonic tissues, indicating potential anti-tumor properties [60]. Therefore, caution should be warranted in interpreting the relationship between *Faecalibacterium* and poor prognostic implications in M-mCRC, as identified in our study.

Our study also unveiled a potential correlation between eight lipid metabolites, potentially influenced by the abundance of the gut microbiome, and M-mCRC-specific survival. We found that genus *Butyrivibrio* and phylum *Proteobacteria* were correlated with reduced levels of 1-oleoyl-GPC (18:1) and 1-palmitoyl-2-dihomo-linolenoyl-GPC (16:0/20:3n3 or 6), respectively. This suggests the involvement of phosphatidylcholine (PC) and its hydrolysis product, lysophosphatidylcholine (LPC), in the gut microbiome’s influence on the host. Studies have demonstrated that an elevated abundance of *Proteobacteria* is associated with metabolic disturbances in the human body, closely linked to inflammatory bowel disease, and may serve as a potential diagnostic indicator for dysbiosis and disease susceptibility [61–63]. Georg et al. identified a significant increase in Proteobacteria abundance in CRC patients by comparing fecal genomes between the CRC and control groups [64]. Further investigation is required to understand the pathogenic mechanisms of Proteobacteria, and our study sheds light on its potential association with lipid metabolism dysregulation. Several studies have demonstrated the correlation between overexpression of lysophosphatidylcholine acyltransferase 1 (LPCAT1), a crucial enzyme influencing PC metabolism and remodeling, with the progression of various malignancies, including CRC [65–67]. Moreover, Kitamura et al. observed elevated levels of lysophospholipids in CRC tissues compared to normal tissues, with LPC, a primary constituent of oxidized low-density lipoprotein, implicated in endothelial dysfunction and potentially contributing to CRC development and progression [68]. Melissa et al. demonstrated that butyric inhibited LPC-induced epithelial barrier dysfunction by enhancing nitric oxide (NO) production derived from neuronal nitric oxide synthase (nNOS) and reducing reactive oxygen species (ROS) generation [69]. Thus, we hypothesize that *Butyrivibrio* may suppress LPC-induced endothelial damage, thereby inhibiting cancer cell invasion and metastasis and ultimately improving the prognosis of M-mCRC.

Moreover, our study revealed a potential role of sphingomyelins (SM) metabolism in mediating gut microbiome’s influence on the prognosis of M-mCRC. MR analysis unveiled a negative association of the abundance of genus *Faecalibacterium* and *Ruminiclostridium*5 with specific SM metabolites, including stearoyl sphingomyelin (d18:1/18:0) and sphingomyelins (d18:2/23:0, d18:1/23:1, and d17:1/24:1). Additionally, *Ruminiclostridium*5 was found to inhibit the levels of SM hydrolysis product ceramides. SM serves as a critical component of cellular membranes, contributing to cellular signaling pathways. Its biosynthesis can modulate the proliferation and apoptosis of cancer cells [70]. SM can be hydrolyzed by sphingomyelinase (SMase) to generate anti-proliferative molecules such as ceramide and sphingosine, as well as pro-proliferative molecules such as sphingosine-1-phosphate [71, 72]. Reduced SMase activity may contribute to aberrant SM metabolism, consequently facilitating the progression of CRC. Notably, Wu et al. found that microbial metabolite butyrate can enhance the activity of acidic SMase in human CRC HT29 cells, promoting the conversion of SM into ceramide [73], suggesting a potential correlation between the gut microbiome and SM metabolism. However, the impact of butyrate-mediated upregulation of acidic SMase expression on tumor development and progression remains to be further studied. Our study revealed a potential correlation between the abundance of butyrate-producing bacterium *Faecalibacterium* and SM metabolites, suggesting that gut microbiome could potentially impact the progression of CRC by acting on the cell membrane, modulating SM metabolism, and inducing aberrant signal transduction pathways. Furthermore, obesity is regarded as a risk factor for CRC. It is postulated that obesity-related dysbiosis of the gut microbiota may increase the risk of cancer [74]. Obesity-associated dysregulation of lipid metabolism has been observed to disrupt intestinal barrier integrity, consequently leading to the induction of metabolic endotoxemia which synergizes with existing adipose tissue inflammation. Intestinal inflammation and toxin-induced DNA damage in intestinal cells are considered potential mechanisms through which dysbiosis of the gut microbiome contributes to the process of carcinogenesis [75, 76]. J et al. identified alterations in the gut microbiome profile among CRC patients with obesity compared to those without obesity. Specifically, there was a reduction in butyrate-producing bacteria and an increase in opportunistic pathogens such as *Prevotella*, *Fusobacterium nucleatum*, *Enterobacteriaceae*, and *Escherichia coli*. These changes may contribute to elevated levels of pro-inflammatory cytokine IL-1β, harmful bacterial metabolite trimethylamine N-oxide (TMAO), and increased intestinal permeability observed in these patients. Thus, it is proposed that the obesity-associated gut microbiome could potentially play a role in the pathogenesis of CRC [77]. Our study provided additional insights into the potential correlation between the gut microbiome and the risk and prognosis of CRC through its impact on lipid metabolism. Nonetheless, further studies are required to validate these observations.

Furthermore, our MR results revealed a positive correlation between the abundance of genus *Olsenella* and three bilirubin degradation products. Additionally, the butyrate-producing bacterium *Butyrivibrio* exhibited a negative correlation with the ratio of Bilirubin (Z, Z) to etiocholanolone glucuronide, indicating a potential suppression of multiple UDP-glucuronosyltransferases (UGTs) corresponding to this ratio. Moreover, these metabolite/metabolite ratios were all associated with worse M-mCRC-specific survival. In hepatic cells, indirect bilirubin (IBI) can be converted to direct bilirubin (DBIL) by binding to glucuronic acid catalyzed by UGT1A1. Elevated levels of total bilirubin (TBIL) and DBIL have been found to be associated with poor survival in patients with NSCLC and CRC. Yang et al. revealed that elevated preoperative levels of TBIL and DBIL were correlated with worse OS in patients with mCRC. They further suggested that DBIL might be an independent prognostic biomarker for mCRC [78, 79]. The gut microbiome could metabolize DBIL into bilirubin derivatives, including urobilin, which can subsequently undergo reabsorption via the hepatic portal vein, thereby modulating signal transmission along the liver-gut axis [80, 81]. We have identified three bilirubin derivatives associated with the prognosis of M-mCRC as potential risk factors and hypothesized that specific gut microbiome could catalyze bilirubin degradation, which in turn affects the gut-liver axis, thereby influencing the metabolism of bilirubin and bile acids. Therefore, further investigation into the interplay of the gut microbiome with bilirubin and its degradation products is crucial for understanding how alterations in bilirubin and bile acid metabolism mediated by gut microbiome contribute to the poor prognosis of M-mCRC. In addition, members of the UGT1A, UGT2A, and UGT2B families predominantly catalyze glucuronidation. Previous investigations have shown that extensive UGT polymorphisms in UGT1A and UGT2B genes were associated with the risk of various malignancies [82, 83]. UGT1A1 gene polymorphisms are common in CRC and have been linked to diminished response rates to irinotecan-based chemotherapy regimens and adverse prognostic outcomes in patients with mCRC [84, 85]. Our study revealed that polymorphisms in several UGTs, including UGT1A1, might be influenced by specific gut microbiomes, suggesting that various potential UGT polymorphisms might affect bilirubin metabolism in M-mCRC patients, thereby potentially inhibiting chemotherapy efficacy and contributing to poor prognoses. Further research is warranted to validate and explore the impact of these polymorphisms in UGTs on M-mCRC.

Despite these significant findings, our study is still subject to several limitations. Firstly, the limited SNPs associated with gut microbiome features lead to a scarcity of IVs, potentially compromising the accuracy of exposure-outcome effect estimates. Secondly, there are limited GWAS studies of survival data in M-mCRC patients. This prevents us from validating our findings regarding gut microbiome and metabolite/metabolite ratios across diverse population cohorts. Third, although mutations in both RAS and BRAF genes can lead to abnormal activation of the MAPK signaling pathway, there are currently no GWAS specifically targeting mCRC patients with individual RAS or BRAF mutations. As a result, we cannot further analyze whether there are clinical prognostic differences in mCRC patients with RAS and BRAF mutations due to changes in the gut microbiome and metabolites. Therefore, we advocate for future genomic studies focusing on mCRC patients with individual RAS and BRAF mutations to estimate the correlation between genetic variants and mCRC-specific survival. This will facilitate comprehensive research involving multi-omics approaches, including microbiomics, proteomics, and metabolomics, in relation to mCRC risk, prognosis, and therapeutic targets. Such research could be helpful for more detailed pathogenic mechanisms and therapeutic strategies, ultimately improving the overall prognosis for these patients.

In conclusion, our study identified associations of gut microbiome and metabolites with the prognosis of M-mCRC. These findings hold significant promise in guiding the exploration of potential therapeutic targets and intervention strategies. Further studies on the microbiome and metabolomics of M-mCRC are warranted.

## Publisher’s Note

All claims expressed in this article are solely those of the authors and do not necessarily represent those of their affiliated organizations, or those of the publisher, the editors and the reviewers. Any product that may be evaluated in this article, or claim that may be made by its manufacturer, is not guaranteed or endorsed by the publisher.

## Supplementary Information

Below is the link to the electronic supplementary material.

Supplementary file1 (PDF 2066 KB)

Supplementary file2 (XLSX 463 KB)

## Data Availability

The original contributions presented in the study are included in the article/Supplementary Material, further inquiries can be directed to the corresponding author/s.

## Abbreviations

CRC: Colorectal cancer
MAPK: Mitogen-activated protein kinase
mCRC: Metastatic colorectal cancer
EGFR: Epidermal growth factor receptor
MSS: Microsatellite stable
M-mCRC: Mutated-RAS/BRAF metastatic colorectal cancer
MR: Mendelian randomization
SNP: Single nucleotide polymorphism
GWAS: Genome-wide association study
IV: Instrumental variable
OS: Overall survival
CLSA: Canadian Longitudinal Study on Aging
MAF: Minor allele frequency
LD: Linkage disequilibrium
IVW: Inverse variance weighted
HP: Horizontal pleiotropy
HMDB: Human Metabolome Database
TCA: Tricarboxylic acid cycle
O/P: Ornithine to phosphate ratio
P5C: δ-1-Pyrroline5-carboxylate
P5CS: δ-1-Pyrroline-5-carboxylate synthase
OTC: Ornithine carbamoyltransferase
NRP-1: Neuropilin-1
LRS: Leucine–tRNA ligase
PC: Phosphatidylcholine
LPC: Lysophosphatidylcholine
SM: Sphingomyelins
IBI: Indirect bilirubin
DBIL: Direct bilirubin
TBIL: Total bilirubin
