# Supplementary material for: Role of blood metabolites in mediating the effect of gut microbiome on the mutated-RAS/BRAF metastatic colorectal cancer-specific survival
Source: Int J Colorectal Dis. 2024 Jul 24;39(1):116. doi: 10.1007/s00384-024-04686-9 (PMC11269474; doi:10.1007/s00384-024-04686-9)
Supplement: Supplementary file 1 — Supplementary file1 (PDF 2066 KB) [file 384_2024_4686_MOESM1_ESM.pdf]

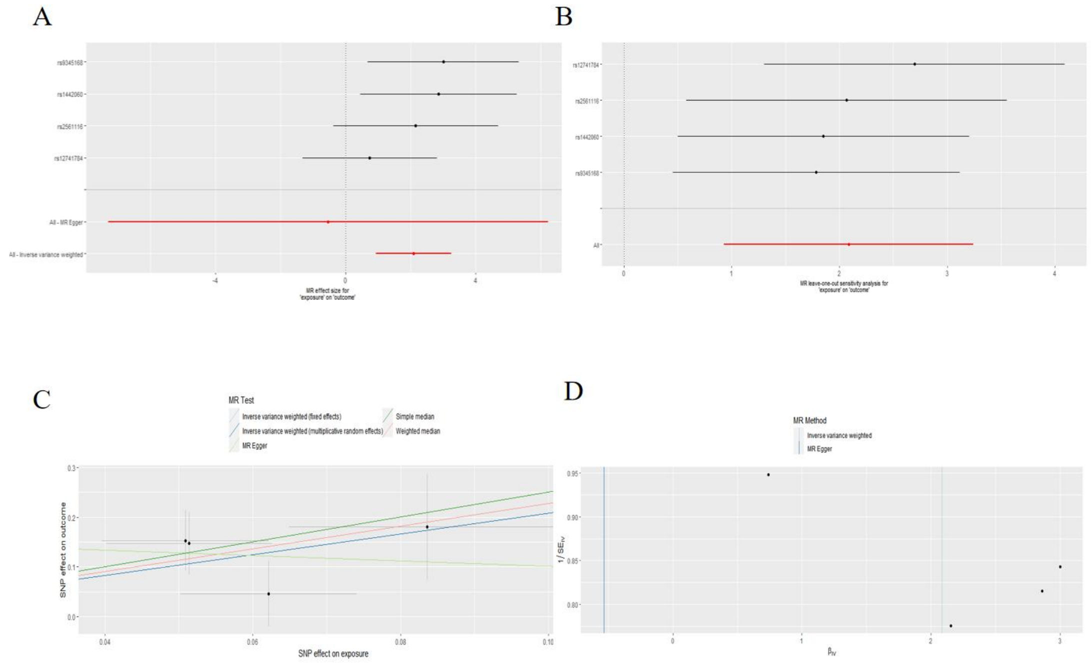

Supplementary Figure 1. Forest plot (A), sensitivity analysis (B), scatter plot (C), and funnel plot (D) of the causal effect of family *V*eillonellaceae.id.2172 on M-mCRC-specific survival.

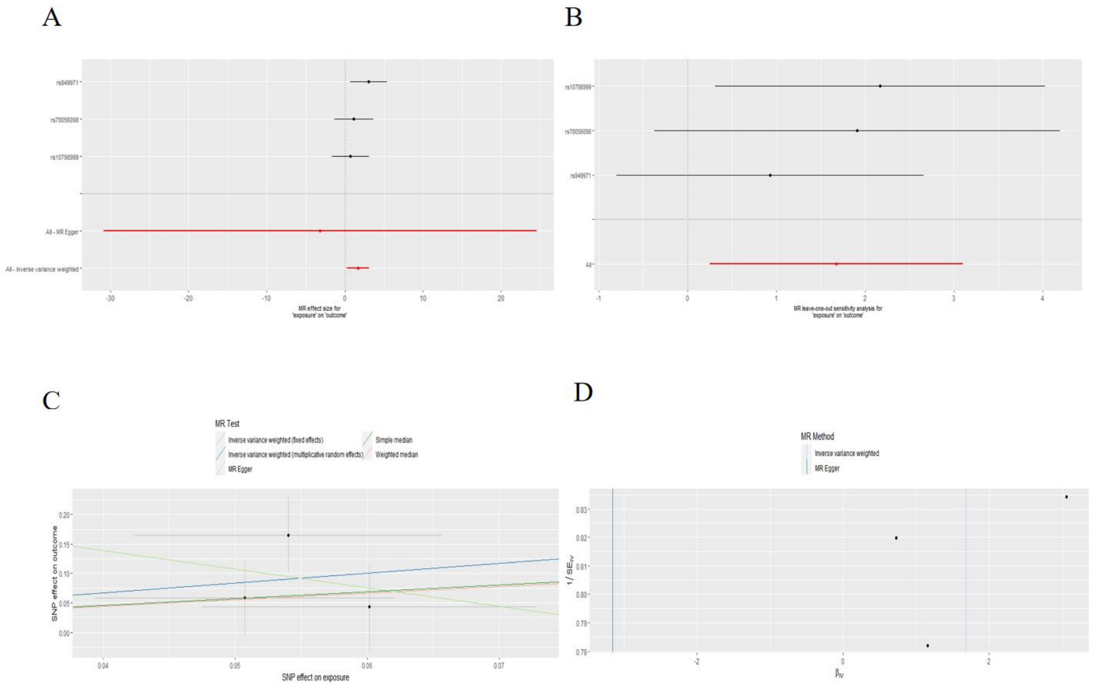

Supplementary Figure 2. Forest plot (A), sensitivity analysis (B), scatter plot (C), and funnel plot (D) of the causal effect of genus *E*ubacteriumhalliigroup.id.11338 on M-mCRC-specific survival.

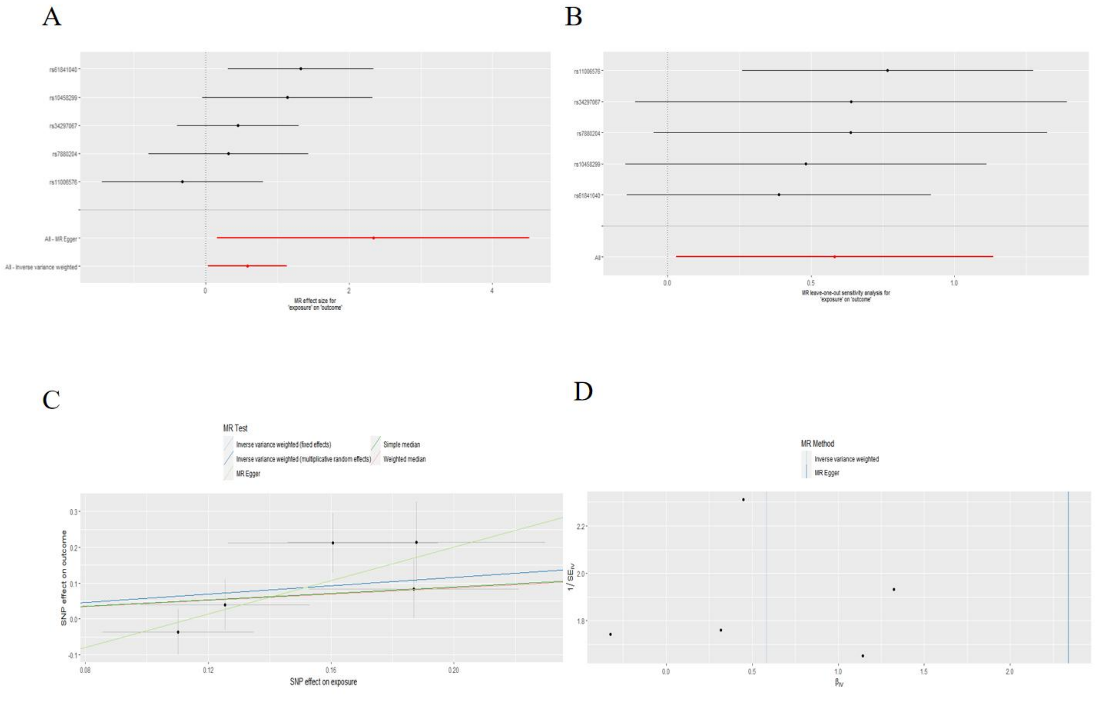

Supplementary Figure 3. Forest plot (A), sensitivity analysis (B), scatter plot (C), and funnel plot (D) of the causal effect of genus *E*ubacteriumnodatumgroup.id.11297 on M-mCRC-specific survival.

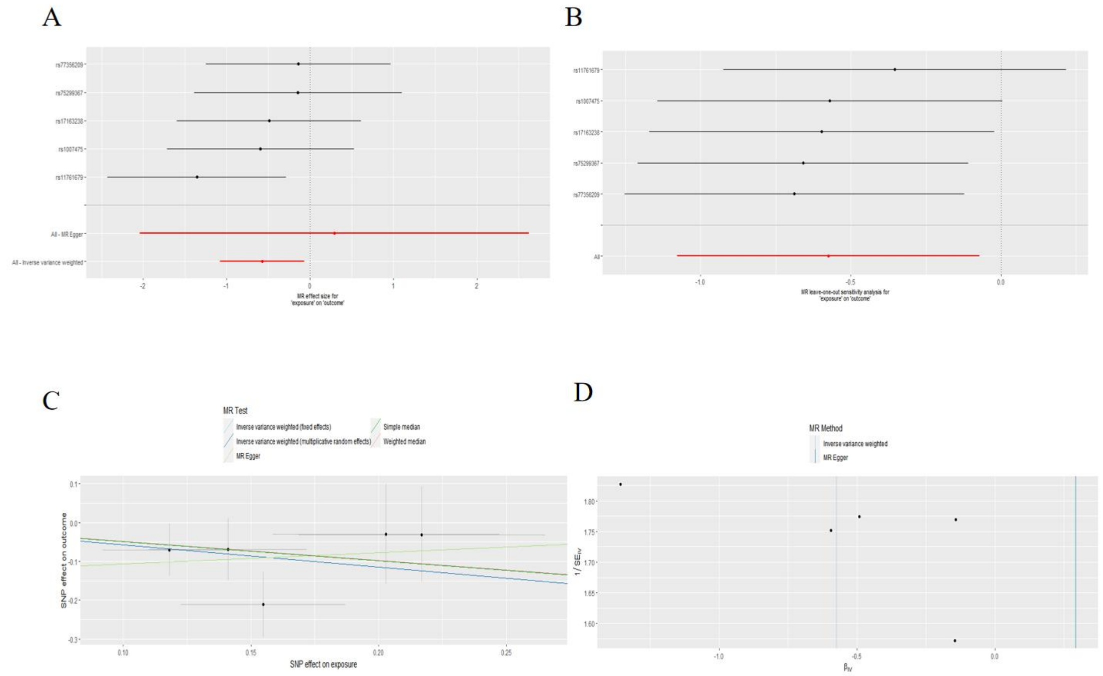

Supplementary Figure 4. Forest plot (A), sensitivity analysis (B), scatter plot (C), and funnel plot (D) of the causal effect of genus *B*utyrivibrio.id.1993 on M-mCRC-specific survival.

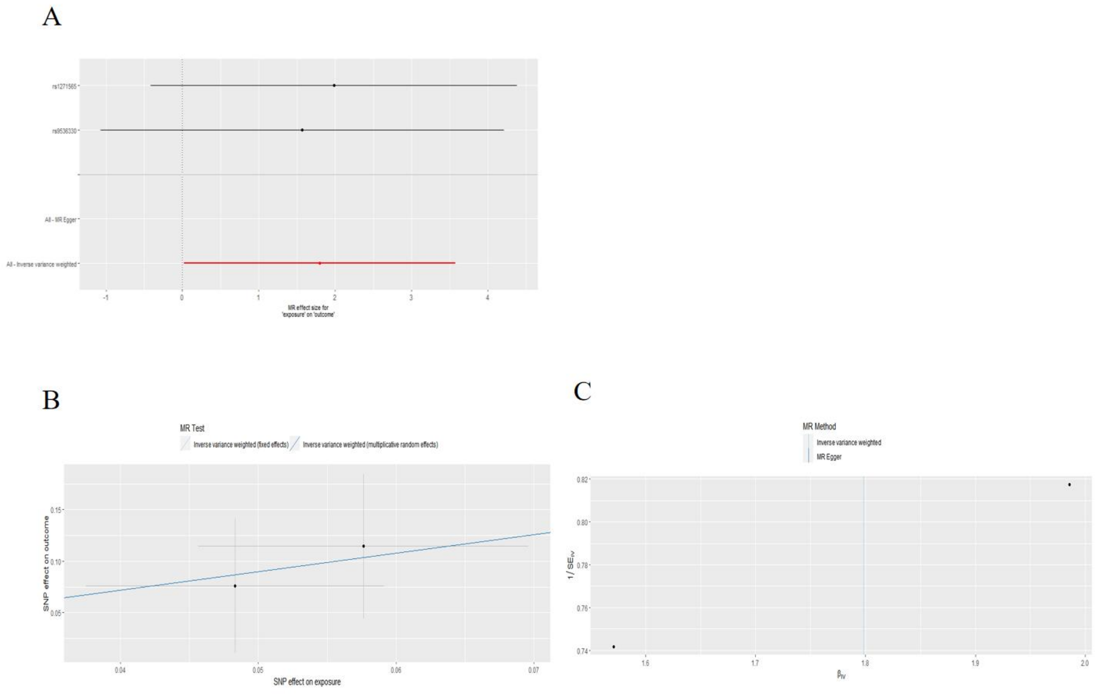

Supplementary Figure 5. Forest plot (A), scatter plot (B), and funnel plot (C) of the causal effect of genus *F*aecalibacterium.id.2057 on M-mCRC-specific survival.

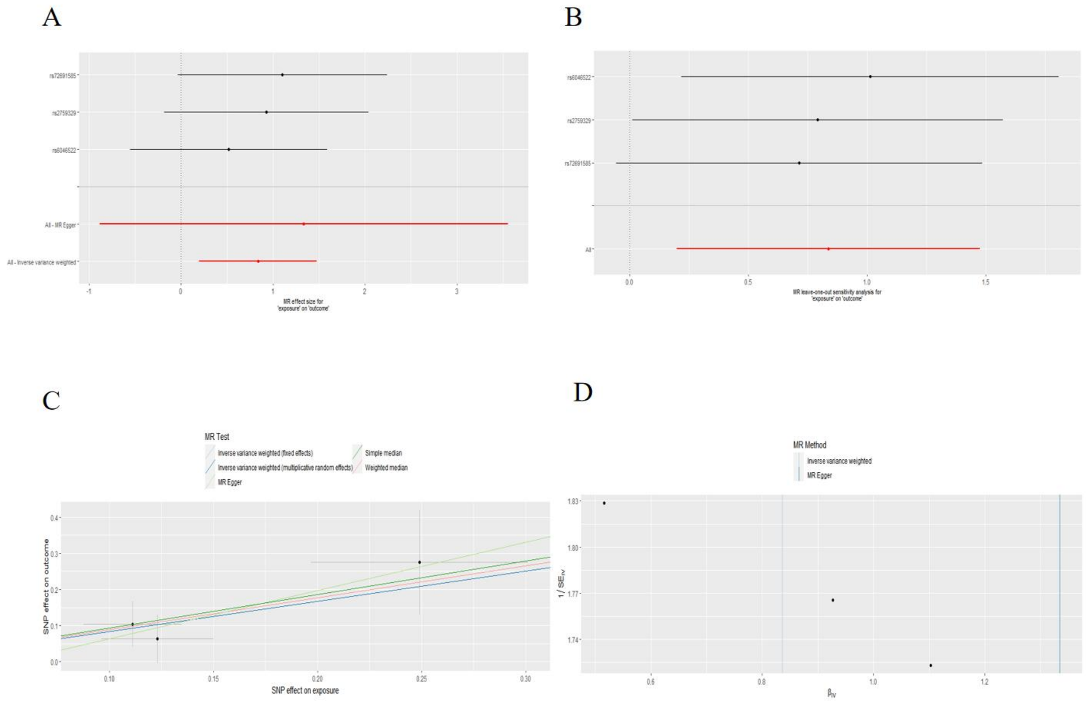

Supplementary Figure 6. Forest plot (A), sensitivity analysis (B), scatter plot (C), and funnel plot (D) of the causal effect of genus *O*lsenella.id.822 on M-mCRC-specific survival.

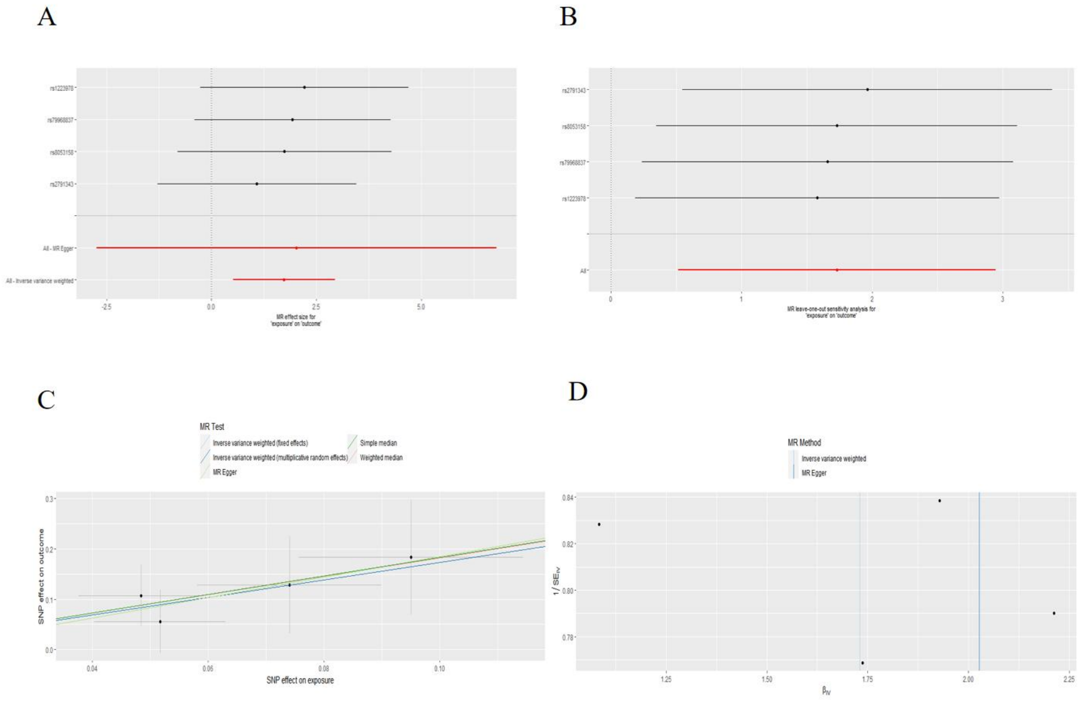

Supplementary Figure 7. Forest plot (A), sensitivity analysis (B), scatter plot (C), and funnel plot (D) of the causal effect of genus *R*uminiclostridium5.id.11355 on M-mCRC-specific survival.

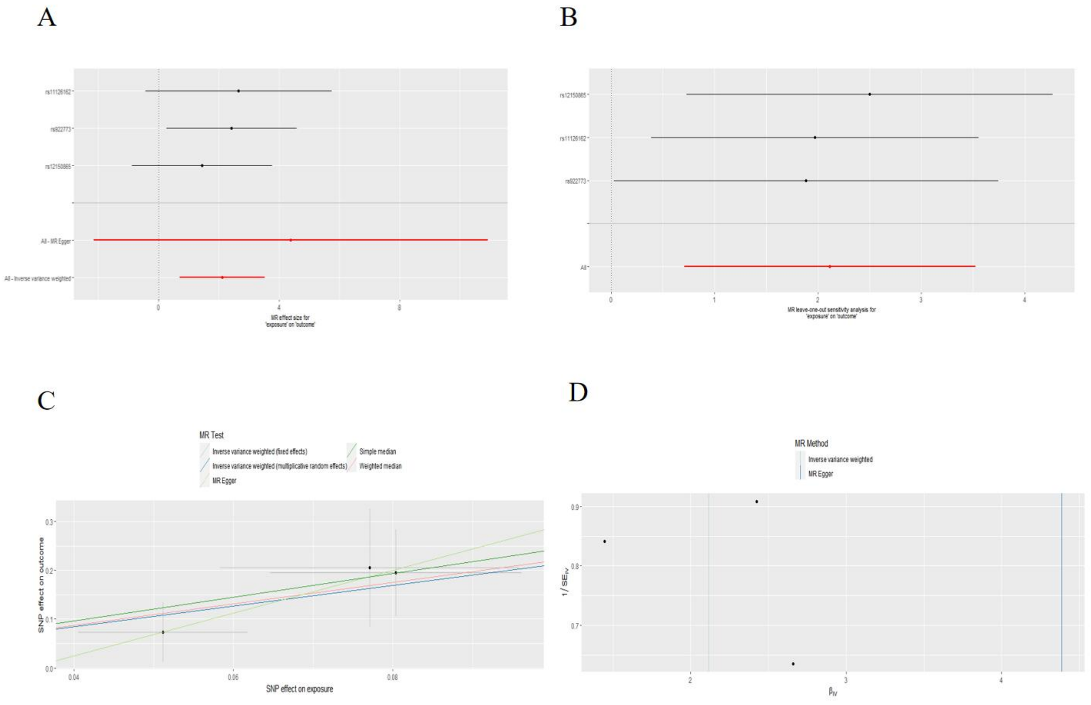

Supplementary Figure 8. Forest plot (A), sensitivity analysis (B), scatter plot (C), and funnel plot (D) of the causal effect of phylum *P*roteobacteria.id.2375 on M-mCRC-specific survival.

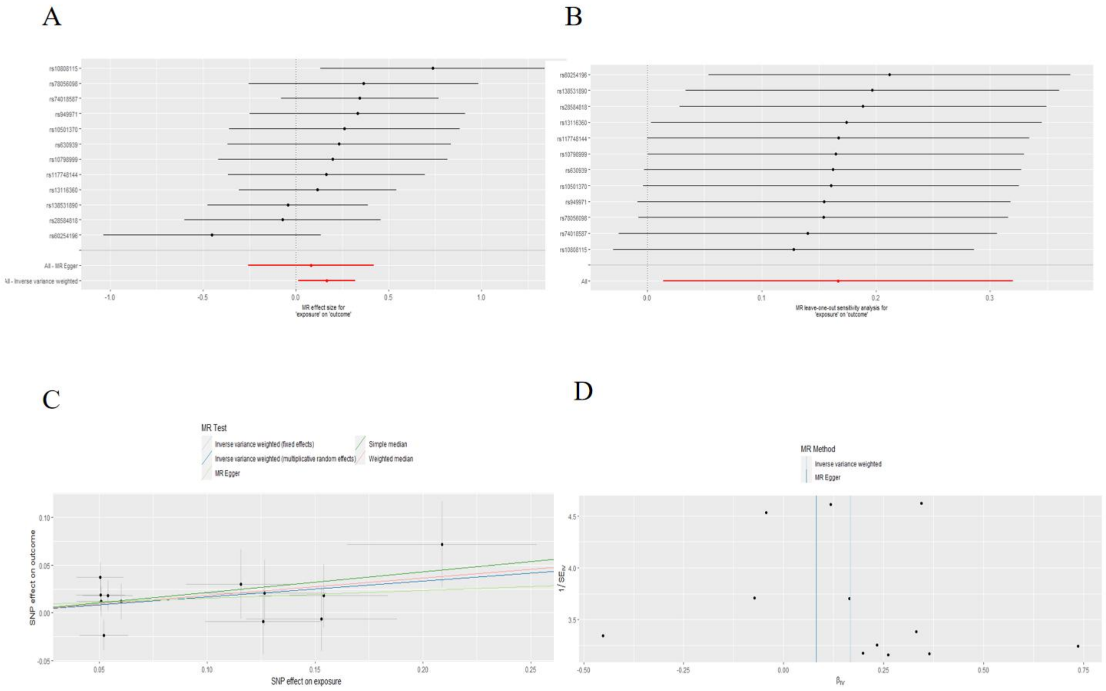

Supplementary Figure 9. Forest plot (A), sensitivity analysis (B), scatter plot (C), and funnel plot (D) of the causal effect of genus *E*ubacteriumhalliigroup.id.11338 on GCST90199716 based on M-mCRC.

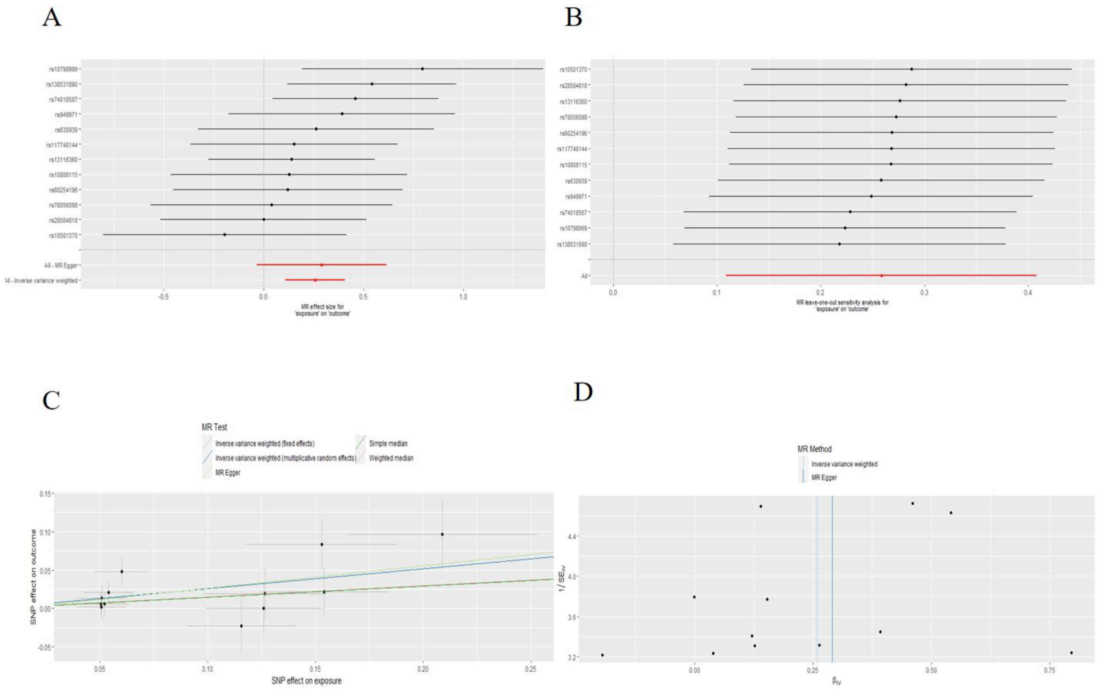

Supplementary Figure 10. Forest plot (A), sensitivity analysis (B), scatter plot (C), and funnel plot (D) of the causal effect of genus *E*ubacteriumhalliigroup.id.11338 on GCST90200901 based on M-mCRC.

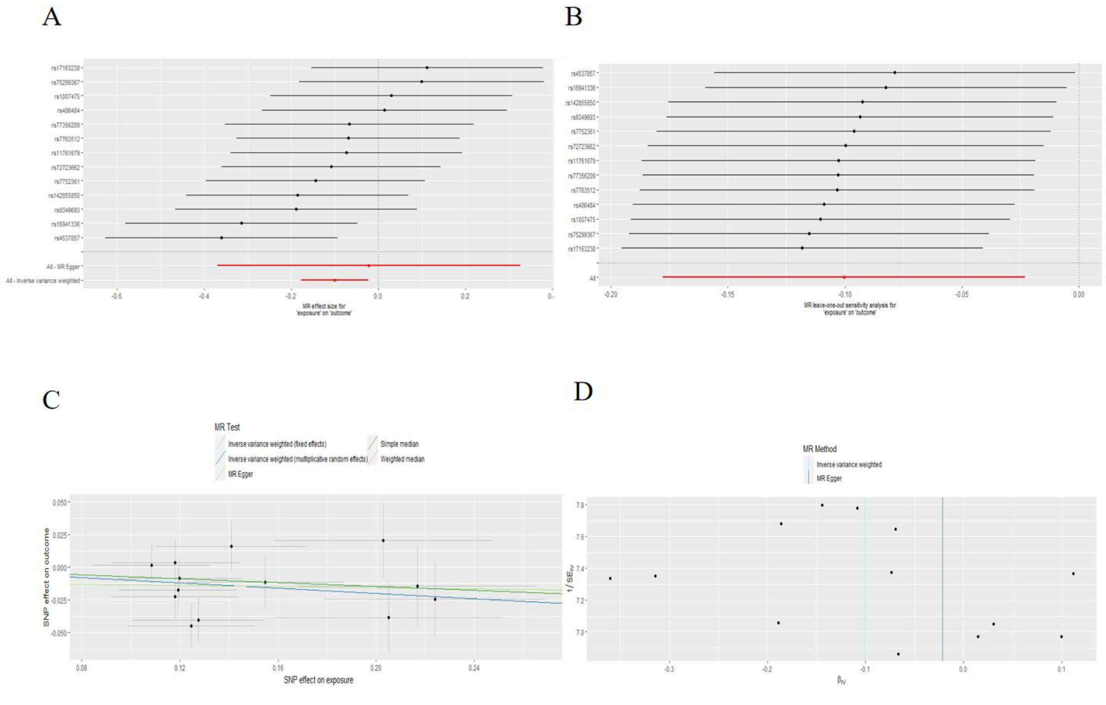

Supplementary Figure 11. Forest plot (A), sensitivity analysis (B), scatter plot (C), and funnel plot (D) of the causal effect of genus *B*utyrivibrio.id.1993 on GCST90199727 based on M-mCRC.

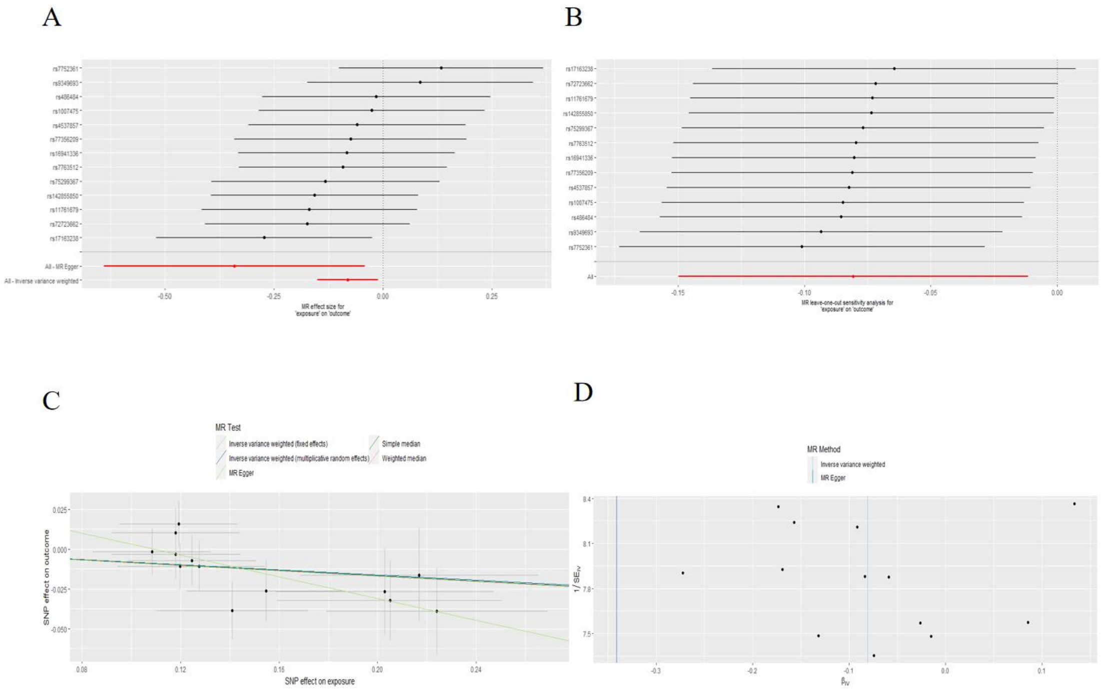

Supplementary Figure 12. Forest plot (A), sensitivity analysis (B), scatter plot (C), and funnel plot (D) of the causal effect of genus *B*utyrivibrio.id.1993 on GCST90200971 based on M-mCRC.

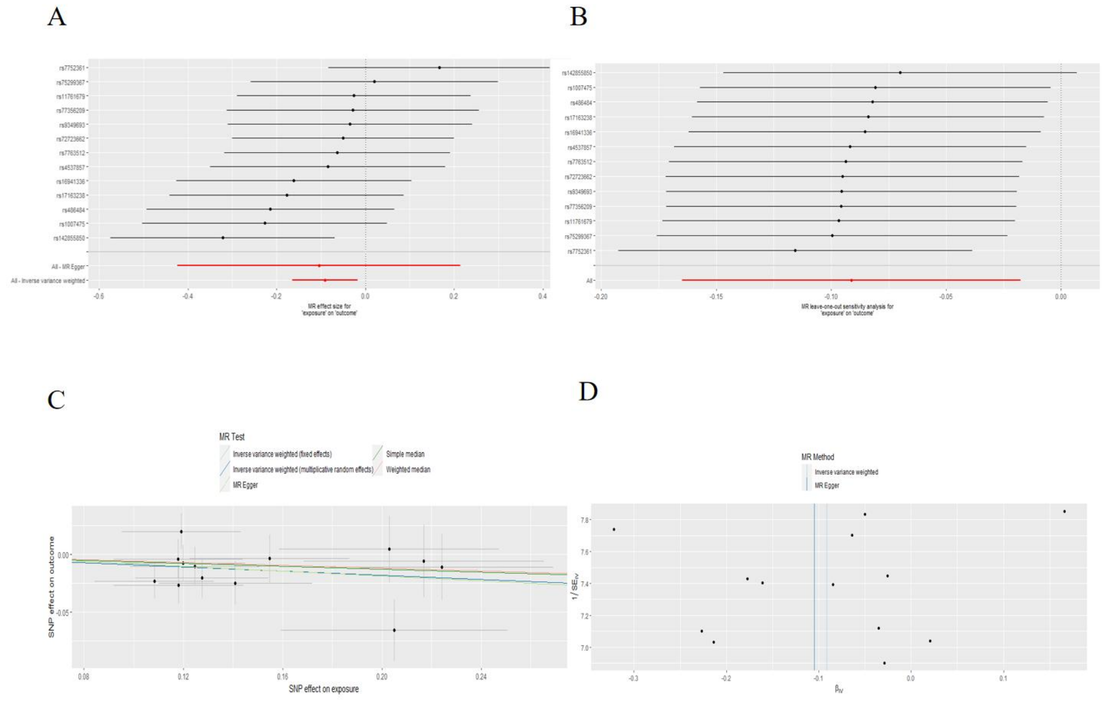

Supplementary Figure 13. Forest plot (A), sensitivity analysis (B), scatter plot (C), and funnel plot (D) of the causal effect of genus *B*utyrivibrio.id.1993 on GCST90201012 based on M-mCRC.

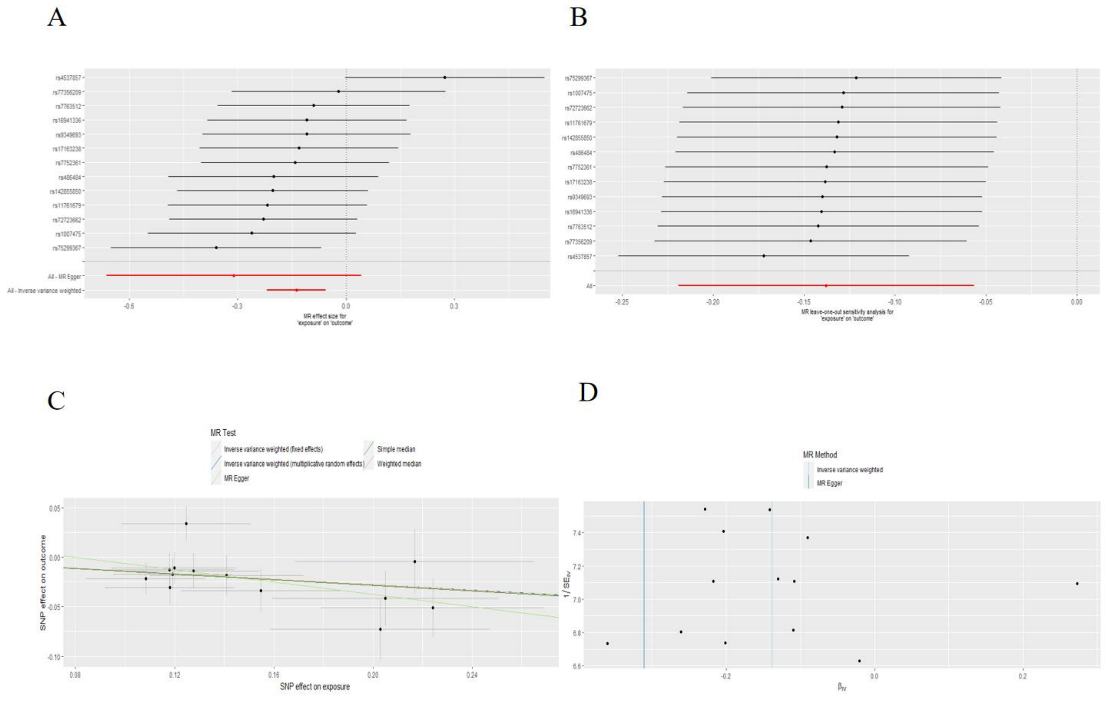

Supplementary Figure 14. Forest plot (A), sensitivity analysis (B), scatter plot (C), and funnel plot (D) of the causal effect of genus *B*utyrivibrio.id.1993 on GCST90201016 based on M-mCRC.

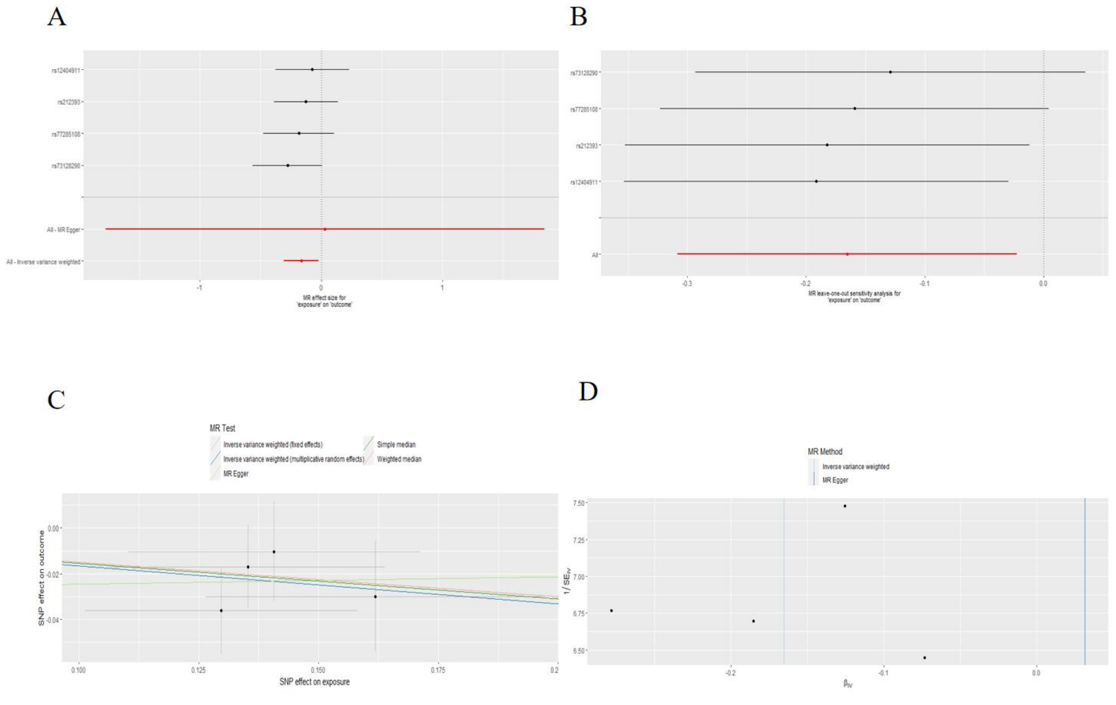

Supplementary Figure 15. Forest plot (A), sensitivity analysis (B), scatter plot (C), and funnel plot (D) of the causal effect of genus *C*atenibacterium.id.2153 on GCST90199790 based on M-mCRC.

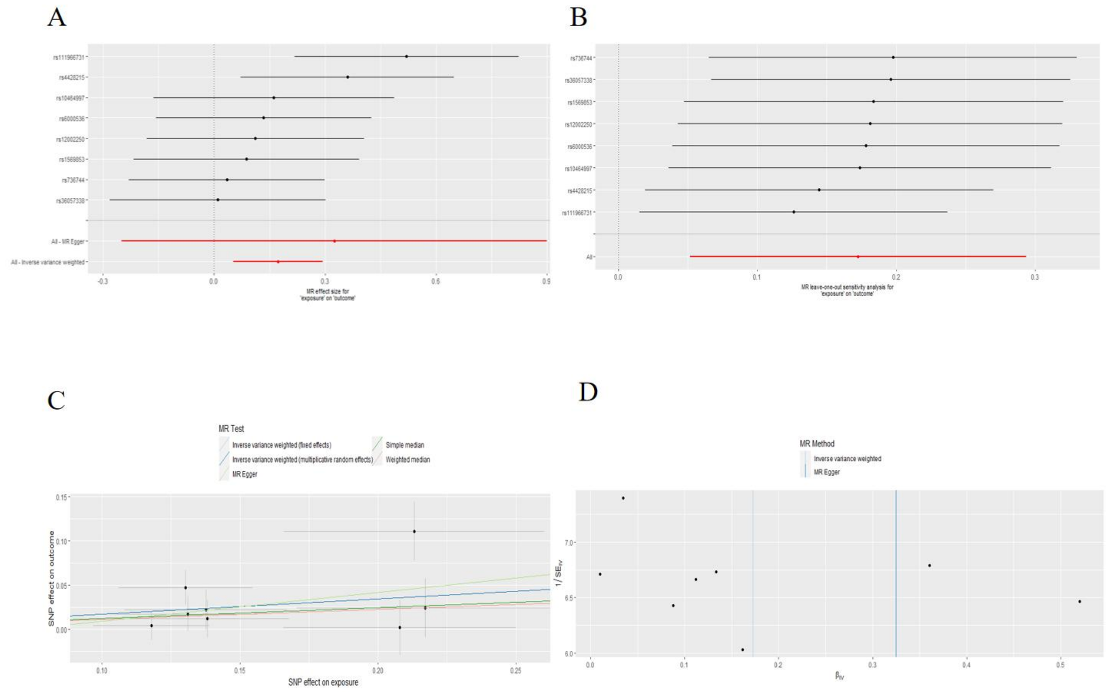

Supplementary Figure 16. Forest plot (A), sensitivity analysis (B), scatter plot (C), and funnel plot (D) of the causal effect of genus *O*xalobacter.id.2978 on GCST90199855 based on M-mCRC.

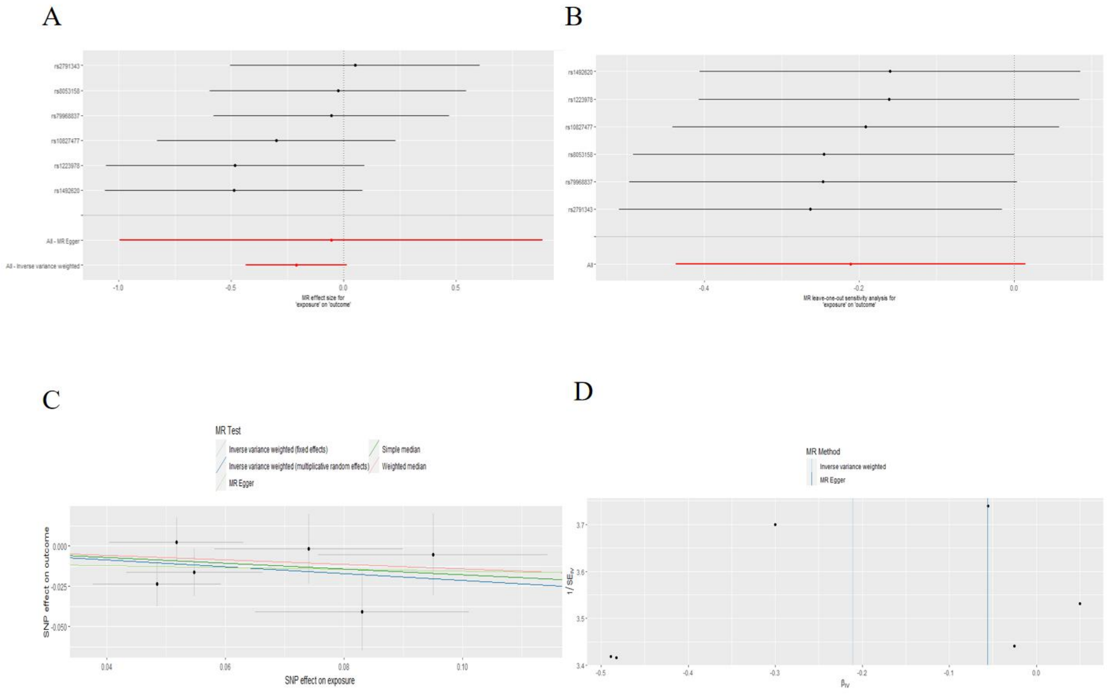

Supplementary Figure 17. Forest plot (A), sensitivity analysis (B), scatter plot (C), and funnel plot (D) of the causal effect of genus *R*uminiclostridium5.id.11355 on GCST90200033 based on M-mCRC.

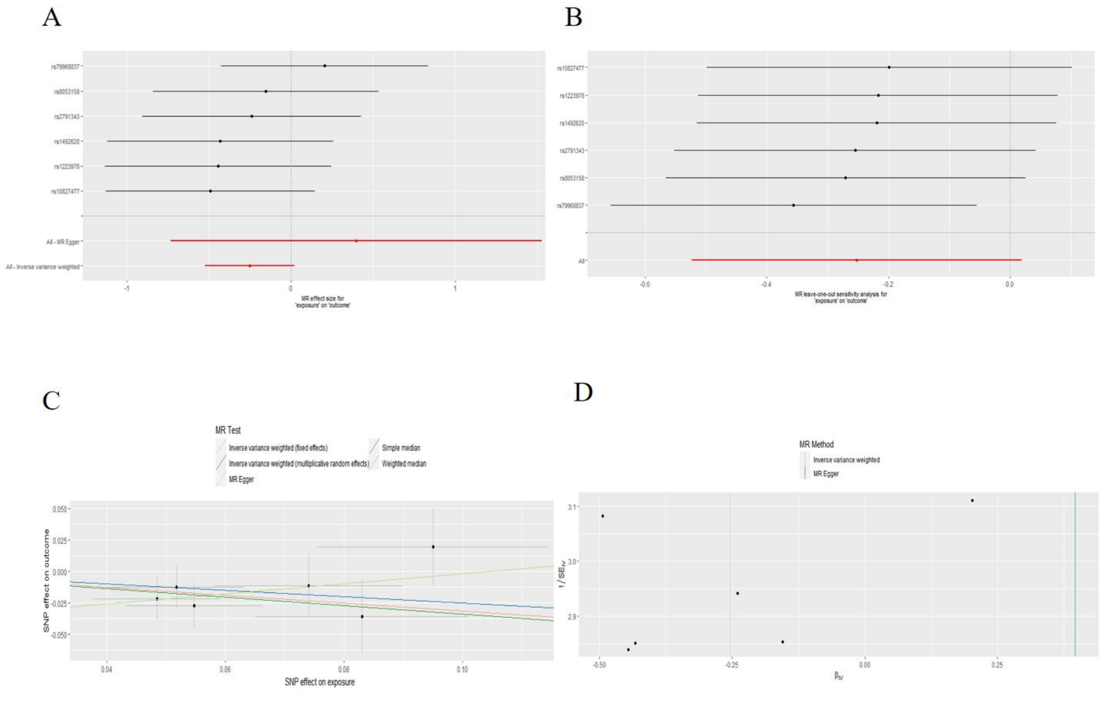

Supplementary Figure 18. Forest plot (A), sensitivity analysis (B), scatter plot (C), and funnel plot (D) of the causal effect of genus *R*uminiclostridium5.id.11355 on GCST90200116 based on M-mCRC.

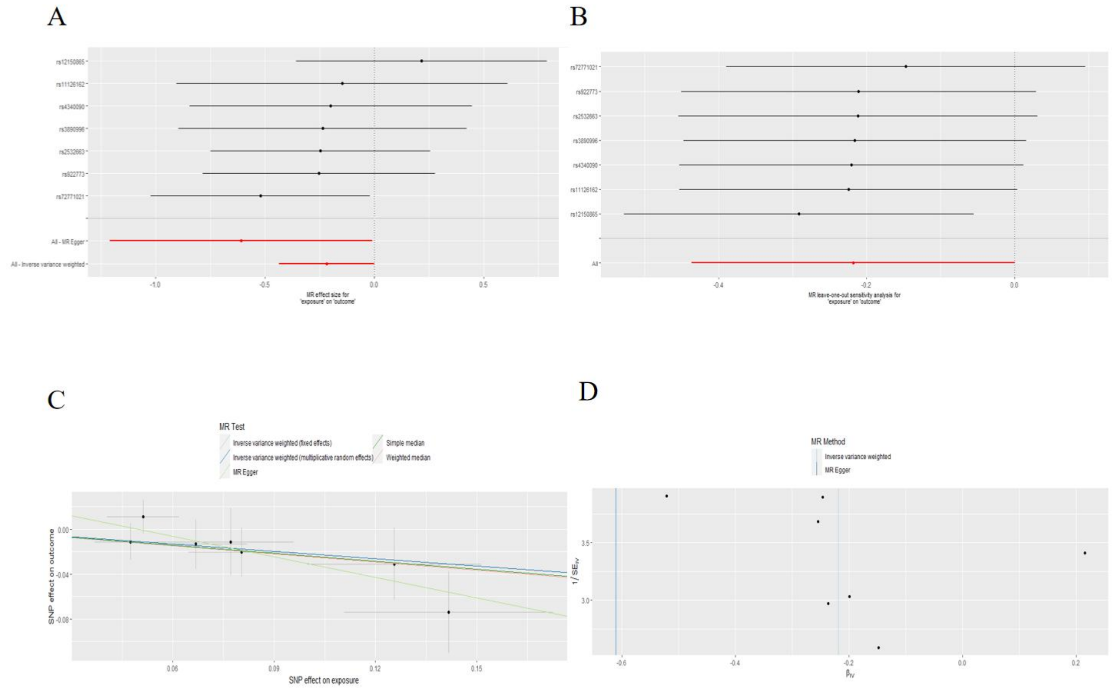

Supplementary Figure 19. Forest plot (A), sensitivity analysis (B), scatter plot (C), and funnel plot (D) of the causal effect of phylum *P*roteobacteria.id.2375 on GCST90200051 based on M-mCRC.

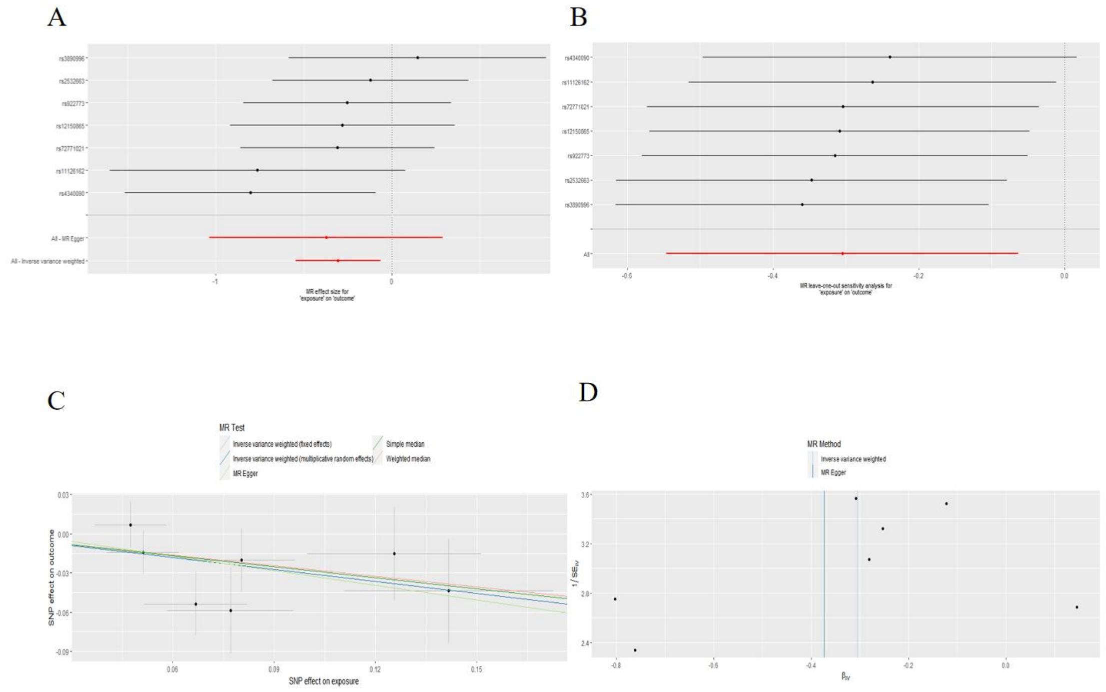

Supplementary Figure 20. Forest plot (A), sensitivity analysis (B), scatter plot (C), and funnel plot (D) of the causal effect of phylum *P*roteobacteria.id.2375 on GCST90200226 based on M-mCRC.

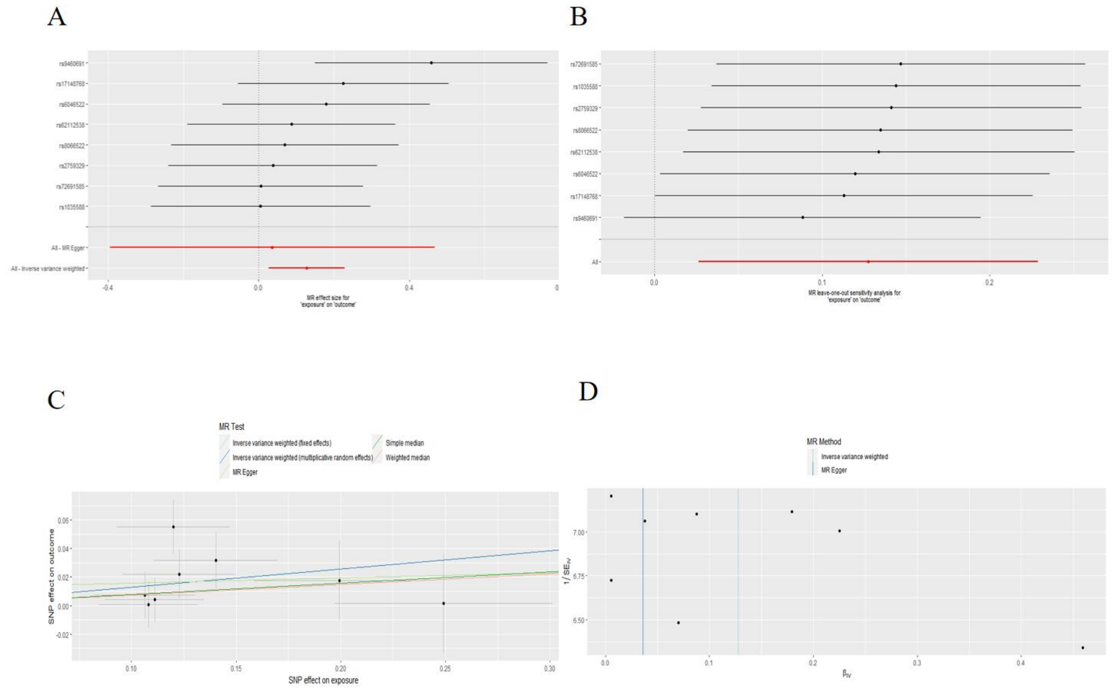

Supplementary Figure 21. Forest plot (A), sensitivity analysis (B), scatter plot (C), and funnel plot (D) of the causal effect of genus *O*lsenella.id.822 on GCST90200264 based on M-mCRC.

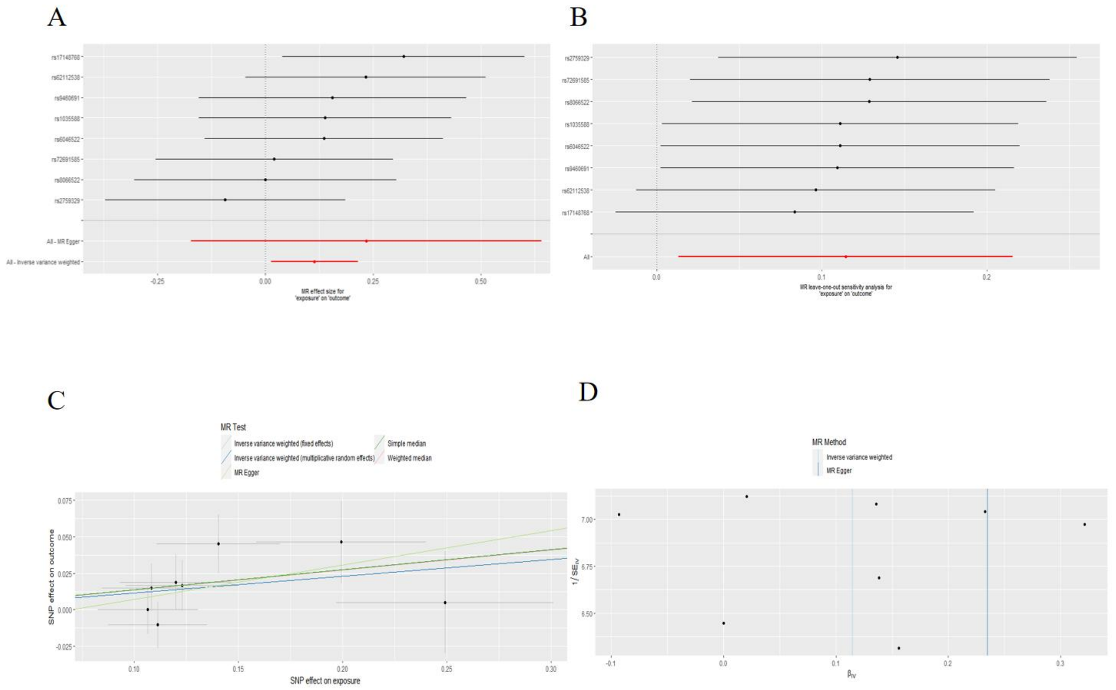

Supplementary Figure 22. Forest plot (A), sensitivity analysis (B), scatter plot (C), and funnel plot (D) of the causal effect of genus *O*lsenella.id.822 on GCST90200265 based on M-mCRC.

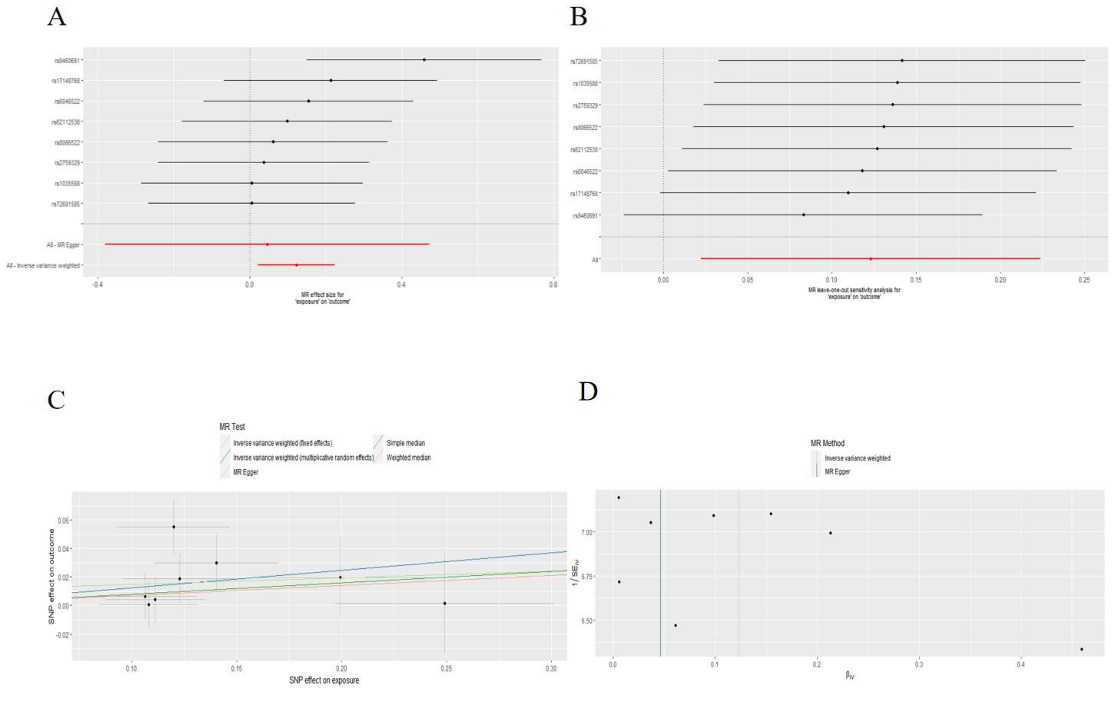

Supplementary Figure 23. Forest plot (A), sensitivity analysis (B), scatter plot (C), and funnel plot (D) of the causal effect of genus *O*lsenella.id.822 on GCST90200269 based on M-mCRC.

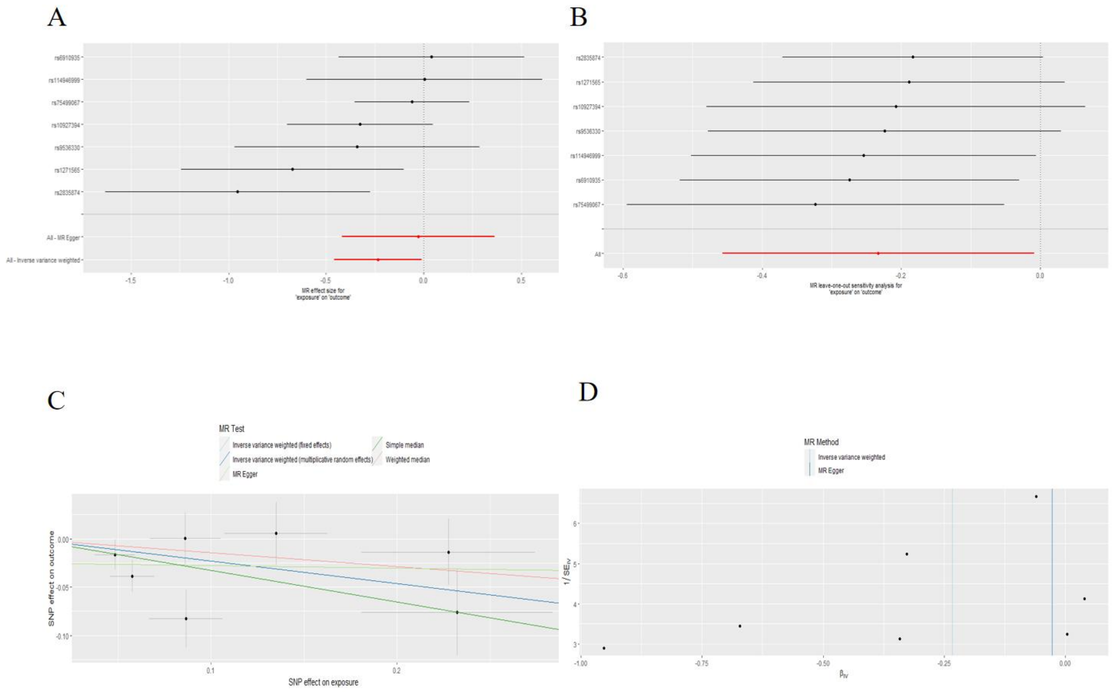

Supplementary Figure 24. Forest plot (A), sensitivity analysis (B), scatter plot (C), and funnel plot (D) of the causal effect of genus *F*aecalibacterium.id.2057 on GCST90200335 based on M-mCRC.

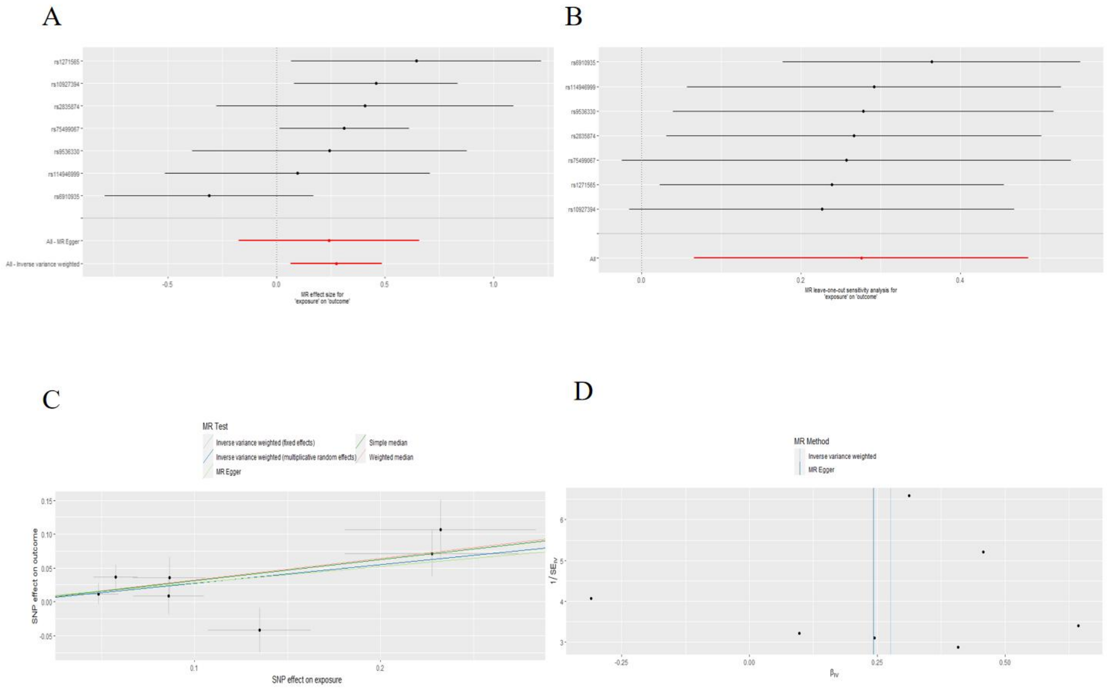

Supplementary Figure 25. Forest plot (A), sensitivity analysis (B), scatter plot (C), and funnel plot (D) of the causal effect of genus *F*aecalibacterium.id.2057 on GCST90201012 based on M-mCRC.
